# Nature-inspired ZnO nanoparticles: unlocking the biomedical potential of *Glycyrrhiza glabra*-mediated green synthesis through *in vitro* and *in silico* approaches

**DOI:** 10.1039/d5ra05670e

**Published:** 2025-09-26

**Authors:** Krishna Kanta Samanta, Manoj Kumar, Himanshu Prasad Mamgain, Pritam Hait, Suvendu Manna, Bibhas Bhunia, Soumen Basu, Jitendra K. Pandey

**Affiliations:** a Department of Chemistry, School of Advanced Engineering, UPES Dehradun Uttarakhand India krishnakanta0096@gmail.com; b Department of Biotechnology, School of Health Sciences and Technology, UPES Dehradun Uttarakhand India manoj.110762@stu.upes.ac.in; c Department of Physics, School of Advanced Engineering, UPES Dehradun Uttarakhand India himanshuhm1111@gmail.com; d Department of Chemistry & Biochemistry, Thapar Institute of Engineering and Technology Patiala Punjab India pritam.kr96@gmail.com soumen.basu@thapar.edu; e Department of Microbiology, Health Sciences Cluster, SOHST, UPES Dehradun Uttarakhand India smanna@ddn.upes.ac.in; f Department of Life Sciences, Parul Institute of Applied Sciences, Parul University Vadodara Gujarat 391760 India bibhasmicro@gmail.com; g HILL Institute, UPES Dehradun Uttarakhand India jeetusnu@gmail.com

## Abstract

In this research work, the aqueous root extract of *Glycyrrhiza glabra* (*G. glabra*) was used to synthesize phytoconstituent-coated zinc oxide nanoparticles (ZnONPs) for the first time. The plant extract served as a reducing and coating/stabilizing agent. The alteration in the physicochemical properties and biomedical potential of the synthesized ZnONPs with an increase in the volume of *G. glabra* extract was investigated, making this study different from others. X-ray diffraction (XRD) analysis confirms that the average crystallite size of the nanoparticles decreased with an increase in the plant extract volume. Field-emission scanning electron microscopy (FESEM) analysis confirmed the formation of uniform spherical particles for all three samples, while energy-dispersive X-ray (EDX) analysis revealed their elemental composition. The samples showed excellent antibacterial activity against Gram-positive *Streptococcus mutans* and Gram-negative *Escherichia coli* with the highest zone of inhibition values of 19.33 ± 0.47 and 25 ± 0.81 mm, respectively. *In silico* molecular docking studies were also performed against two different receptors, *i.e.*, DNA gyrase B (*E. coli*) and antigen-I/II carboxy-terminus (*S. mutans*) proteins with several phytoconstituents (identified through gas chromatography-mass spectroscopy (GC-MS) analysis). Among the four phytoconstituents, 9,12-octadecadienoic acid (*Z*,*Z*)- (−5.5, and −4.8 kcal mol^−1^) and *n*-hexadecanoic acid (−5.5 and −4.5 kcal mol^−1^) exhibited the highest binding affinity. The molecular docking outcome demonstrates good agreement with the *in vitro* result. Additionally, the cell viability of the as-synthesized ZnONPs against a normal cell line (HaCaT) was >95% compared to the cell viability against cancer cells (69.5% ± 3.8%), which indicates that the sample has higher selectivity towards cancer cells. Subsequently, the minimal toxicity of the phytochemical-coated ZnONPs enhances their suitability across diverse biomedical fields, especially in combating bacterial infections.

## Abstract 2

This study focused on the green synthesis of phytochemical-coated ZnONPs for biomedical applications. Standard *in vitro* assay confirms excellent antibacterial potential of ZnONPs, which was well supported by the *in silico* study.

## Graphical abstract

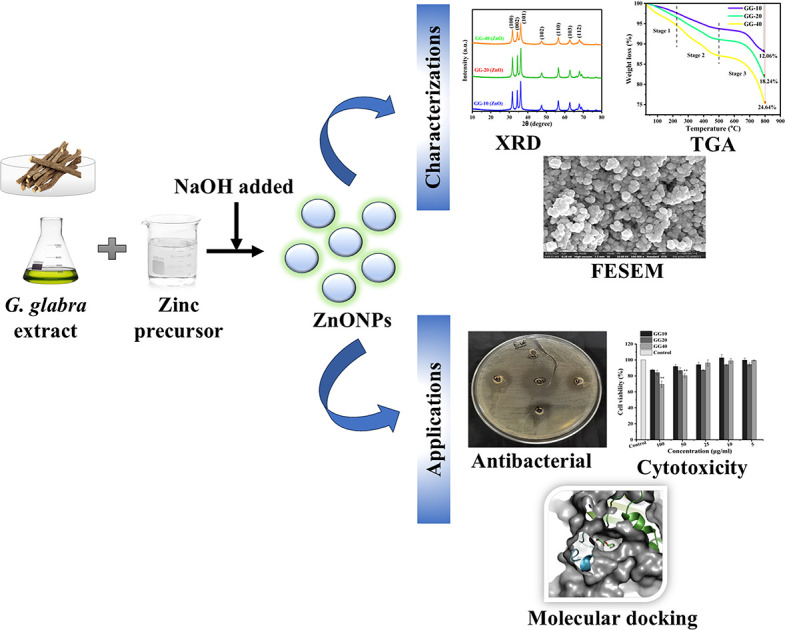

## 1. Introduction

The discovery of antibiotics is a major and significant achievement in the field of medicine. They have become indispensable in front-line medical procedures such as surgery, organ transplantation, and treating numerous bacterial infectious diseases. Unfortunately, the misuse of antibiotics has led to the development of antibiotic resistance in bacterial species.^1,2^ Consequently, their therapeutic efficacy is currently failing due to the enormous rise in bacterial resistance to antibiotics. Considering this, plant-based metallic nanoparticles are an ideal replacement for existing antibiotics and show great promise in addressing the issue of the emergence of bacterial multidrug resistance (MDR).^3^ Among the various types of monometallic nanoparticles (MNPs), bimetallic nanoparticles (BNPs), and metal oxide nanoparticles (MONPs), MONPs are considered promising materials for pharmacological applications due to their high stability; ability to be engineered to the desired sizes, morphologies, and porosities; ease of incorporation into hydrophobic and hydrophilic systems; and susceptibility to crosslinking by different molecules owing to their negatively charged surface.^4,5^ Therefore, they have been viewed as an important and widely distributed area of physiological study encompassing antiprotozoal,^6^ antimalarial,^7^ anti-inflammatory,^8^ antimicrobial,^9^ antioxidant,^10^ antidiabetic,^11^ and anticancer therapeutics.^12^ Subsequently, among the various MONPs, zinc oxide nanoparticles (ZnONPs) have garnered significant interest in recent years. This is attributed to their remarkable optical, electrical, mechanical, and pharmacological properties, coupled with their low toxicity and high biocompatibility. The US FDA classified ZnO as a material that is “generally recognized as safe” (GRAS). Currently, ZnONPs are widely used in various applications, including catalysis, semiconductors, textiles, cosmetics, chemical sensing, healthcare, and food packaging.^13^ The healthcare applications of ZnONPs include anticancer,^14^ anti-fungal,^15^ anti-inflammatory,^16^ antidiabetic and antibacterial activities.^17^ In the last few decades, ZnONPs have been produced through various methods, namely, physical, chemical, and biological routes. Nonetheless, physical methods entail higher energy demands and the requisite for sophisticated instrumentation, thus presenting logistical hurdles. Conversely, chemical methods employ expensive and potentially hazardous reducing agents such as sodium borohydride (NaBH_4_), thiols, and amines, impeding their scalability, economic feasibility, and environmental compatibility. Thus, to address these challenges, natural resources such as plants and microbes are harnessed for the environmentally friendly and cost-effective production of metallic nanoparticles.^18^ However, plants are preferred over microbial sources for synthesizing metallic nanoparticles due to their local availability, ease of handling, and lower costs. The extraction of mycochemicals (which act as reducing and capping agents in green synthesis) from microorganisms including bacteria and fungi incurs an additional culturing cost and is time-consuming.^19^ In this regard, many researchers have synthesized ZnONPs using different plant extracts to date, *e.g. Psidium guajava*,^20^*Thymbra spicata* L.,^21^*Phoenix roebelenii*,^22^*Myristica fragrans*,^23^*Parthenium hysterophorus*,^24^*Ruellia tuberosa*,^25^ and *Cassia auriculata*,^26^ for biomedical applications. However, limited research has been done on how different quantities of phytochemicals used as reducing/capping agents during the manufacturing process of nanoparticles affect their antibacterial activity and cytotoxicity towards both cancerous and normal cell lines.

*Glycyrrhiza glabra*, commonly known as mulethi or Yastimadhu, is an evergreen shrub that is native to India, the Middle East, and some regions of Africa. In India, it grows naturally in the hilly regions of Uttarakhand, Himachal Pradesh, and different states of South India such as Andhra Pradesh, Karnataka, and Tamil Nadu, whereas it is cultivated in eastern states such as Odisha and West Bengal. This plant contains several classes of medicinally important secondary metabolites such as coumarin, flavonoids, polysaccharides, alkaloids, saponins, and stilbenes,^27,28^ which has culminated in the rigorous study of plant-based ingredients for the synthesis of MONPs.^29^ Many previous studies reported that *G. glabra* root extract has significant antimicrobial activity against several bacteria such as *E. coli*, *S. mutans*, and *S. aureus*.

In light of the aforementioned information, the primary goal of this study was to synthesize ZnONPs in an eco-friendly and cost-effective manner instead of using harmful and expensive reducing and capping/stabilizing agents. In addition, the synthesis procedure was optimized by considering the reaction time for three different volumes of plant extract (*i.e.* 10, 20, and 40 mL). According to the literature, no study has been reported to date on the biosynthesis of ZnONPs using *G. glabra* root aqueous extract. Finally, the *in vitro* antibacterial activity of the as-synthesized ZnONPs was tested against Gram-positive *Streptococcus mutans* (a cavity-causing bacterium) and Gram-negative *Escherichia coli* (a common bacterium that is responsible for nosocomial infections). Furthermore, a molecular docking study was performed using different bioinformatics tools against DNA gyrase B (*E. coli*) and antigen-I/II carboxy-terminus (*S. mutans*) to determine the binding affinity of each phytoconstituent (present in the prepared *G. glabra* extract) and standard antibiotics. An *in vitro* cytotoxic study was done to check the toxic effect of the as-synthesized ZnONPs against normal human skin (HaCaT) and cancer (HepG2) cell lines. A comparative cytotoxic study was also performed among the existing reports on ZnONPs produced through chemical/green synthesis methods with this study.

## 2. Materials and methods

### 2.1. Materials

The chemicals employed in this research work including sodium hydroxide (NaOH, ≥98%), zinc nitrate hexahydrate (Zn(NO_3_)_2_·6H_2_O, ≥99%), ethanol (EtOH, ≤99.9%), methanol (MeOH, ≤99.9%), and Mueller–Hinton agar powder were purchased from Merck, India. Milli-Q water was used to prepare all solutions. The chemicals used during this work were of analytical (AR) grade.

### 2.2. Collection and preparation of *G. glabra* extract

The dried roots of *G. glabra* were obtained from a local market (Dehradun, Uttarakhand, India). The roots were washed with Milli-Q water several times to remove dust particles and left to dry in an oven at ∼40 °C overnight (approximately 12 h). Then, the dried roots were grounded into a uniform powder using an electrical grinder. Finally, 10 g of root powder was mixed in 100 mL Milli-Q water (10% (w/v)) and left soaking overnight. After 16 h, the solution was allowed to boil at ∼40 °C for 120 min at 300 rpm (revolutions per minute). Then, the mixture solution (having a brownish colour) was cooled and filtered using Whatman no. 1 filter paper. The pH of this extract solution was about ∼4.88, which was stored at −4 °C for further work. This solution was directly utilized for the synthesis of ZnONPs without further concentration or drying. However, to determine the percentage (%) yield of the crude plant extract, the aforementioned extraction method (decoction) was repeated using 10 g of root powder, and the final extract solution was dried overnight in a hot air oven at 60 °C ± 2 °C. The percent yield extraction from *G. glabra* was calculated using the following equation:
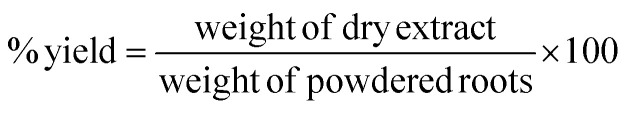


Similarly, 5 g of root powder was mixed with 50 mL of methanol and heated at ∼40 °C to prepare the methanolic extract. The gas chromatography-mass spectrometry (GC-MS) analysis technique was performed using the methanol extract.

### 2.3. Synthesis of ZnO nanoparticles

The general procedures followed to synthesize all the ZnONP samples following a previously published work with slight modification^30^ (only altering the volume of extract) are shown in Fig. S1a. Mainly, Zn(NO_3_)_2_·6H_2_O was dissolved in 90 mL Milli-Q water and the solution (having concentration 0.1 M) was kept on a hot plate magnetic stirrer (Borosil HLS-200) to obtain a homogenous mixture of zinc salt solution. After about 5 min, 10 mL of plant extract was added to this solution. Subsequently, 1.0 M of NaOH was added dropwise to maintain pH ∼9.0, and the resulting solution was continuously stirred for 2 h at 40 °C and 700 rpm. The sample solution was collected at regular intervals spanning from 30 min to 2 h for time optimization. The formulation of the nanoparticles was monitored and confirmed visually by the change in color of the mixture. A white to yellowish color in the nanoparticle solution was observed at different time gaps, as shown in Fig. S1b. Then, the mixture was cooled at room temperature and centrifuged using a REMI C-24 plus centrifuge at 12 000 rpm for 15 min. The supernatant was discharged, and the precipitate was collected. This aforementioned step was repeated two times by adding primarily Milli-Q water, and then 50% (v/v) ethanol with the collected precipitate to remove all the undesired products. The final product was then dried in a hot-air oven at 40 °C overnight and stored in an airtight sample bottle for further characterization and biological studies. The as-synthesized ZnONP samples were marked as GG-10, GG-20, and GG-40 for 10, 20, and 40 mL of plant extract, respectively. In this study, the temperature did not exceed 40 °C at any stage of the experimental process to retain a good amount of phytoconstituents on the nanoparticle surface. In another experimental setup, chemically synthesized ZnONPs were prepared by mixing zinc nitrate solution with 0.1 M NaOH without adding plant extract. Other reaction parameters such as medium pH, rotation speed, incubation time, and drying temperature remained constant. The corresponding sample was marked as Chem.-ZnO.

### 2.4. Material characterization

GC-MS analysis was carried out using a CLARUS SQ8S, PerkinElmer instrument to identify the phytoconstituents present in the prepared plant extract. The conditions for GC-MS analysis were as follows: injection volume = 1 μL, gas (helium) flow rate = 1 mL min^−1^, MS source temperature = 250 °C, and GC-MS transfer line temperature = 250 °C. The phytochemicals were identified from the GC-MS mass spectrum by comparing the data of known compounds with the NIST library. UV-visible spectroscopy measurements (200–800 nm) were performed using a double-beam spectrophotometer (UV-1900 UV-VIS spectrophotometer, Shimadzu) to ensure the formation of ZnONPs. The absorption maximum (*λ*_max_) of the samples was recorded using a quartz cuvette (path length: 1 cm). In our study, the direct band gap (*E*_g_) of all the as-prepared samples was obtained using the UV-visible absorption data through Tauc's equation, *i.e.*, eqn (1),^31^ as follows:

1 *αhν* = *k*(*hν* − *E*_g_)^*n*^

where *α* is the absorption coefficient, *hν* is energy of a photon, *k* is a constant, and *E*_g_ is the band gap energy (in eV). The value of ‘*n*’ changes with the electronic transitions, and it can be 1/2, 2/3, 2, or 3 for indirect-allowed, direct-forbidden, direct-allowed, and indirect-forbidden transitions, respectively. Given that ZnO is primarily considered a direct-band gap material, the value of ‘*n*’ is 2.

The attachment of functional groups on the surface was identified using Fourier transform infrared (FTIR) spectroscopy (Frontier FT-IR/FIR, PerkinElmer) with the KBr pellet method over the wavenumber region of 450 to 4000 cm^−1^. The thermal stability and quantity of phytoconstituents that were coated on the surface of ZnONPs were confirmed by thermogravimetric analysis (TGA) (NEXTA STA 200, Hitachi, Japan), heated at 25 °C to 800 °C (heating rate = 10 °C min^−1^) in an N_2_-gas atmosphere (gas flow rate = 100 mL min^−1^). The crystallite size (*D*), crystalline nature, and purity of the biosynthesized ZnONPs were analyzed using the X-ray diffraction (XRD) technique (D8 ADVANCE ECO-Bruker) equipped with a copper (Cu) target for generating CuK_α_ radiation (*λ* = 1.5406 Å or 0.15406 nm) in the 2*θ* range of 10° to 80°. The average crystallite size was calculated using the Scherrer equation, as given by eqn (2):

2 *D* = *Kλ*/*β* cos *θ*

Moreover, a modified form of the Williamson–Hall method was employed to determine the crystal structure and lattice microstrain (*ε*) using eqn (3), as follows:

3 *β*_*hkl*_ cos *θ* = 4*ε* sin *θ* + *Kλ*/*D*

where *θ* is Bragg's diffraction angle (in radians), *λ* is the incoming X-ray wavelength (0.15406 nm), *D* is the mean crystallite size (in nm), *K* is the Scherrer constant, which usually depends on the crystal shape (the value typically around 0.9), *ε* is the crystal microstrain, and *β* is the full-width at half maximum (in radians) of the XRD peaks. The above-mentioned eqn (3) is similar to the straight-line equation, where (*Kλ*/*D*) is the *y*-intercept and *ε* is the slope.

Additionally, the average crystallite size was calculated using the modified Debye–Scherrer equation, which considers the least squares fit across all the diffraction peaks.

From eqn (2),

4 *β* = *Kλ*/*D* cos *θ*

Taking natural log on both sides of this equation,

5 ln(*β*) = ln(*Kλ*/*D*) + ln(1/cos *θ*)

By plotting ln (*β*) *vs.* ln (1/cos *θ*), we can find a straight line and the intercept of this line was utilized to calculate the crystallite size (*D*).

The size distribution and average hydrodynamic size (*d* nm) of all three time-optimized ZnONP samples were measured *via* dynamic light scattering (DLS) (ZEN1690, Malvern Instruments Ltd, 2013) at a scattering angle of 90°. Milli-Q water was used as the dispersion medium. Zeta potential (*ζ*-potential) was applied to measure the stability and surface charge of the particles using a ZEN 3600, Malvern, U.K. instrument at a temperature of 25 °C. Further, the particle size and morphology of the synthesized ZnONPs were analysed using a field emission scanning electron microscope (FESEM) (FEI, Apreo LoVac), and their elemental composition was assessed using energy-dispersive X-ray spectroscopy (EDX). The instrumental results were graphically plotted using Origin software (OriginPro2023b, learning edition).

### 2.5. Biomedical application of phytochemical-coated ZnONPs

#### 2.5.1. Antibacterial studies of ZnONPs

The standard agar well diffusion assay was used to test the antibacterial activity of ZnONPs against the Gram-negative bacterium *Escherichia coli* (MTCC 2126) and Gram-positive bacterium *Streptococcus mutans* (MTCC 890).^32^ For this, ZnONP powder (about 30 mg) was dispersed in double-distilled water (100 mL) and sonicated for 15 min at 30 °C. The 24 h bacterial culture was spread over MH agar plates and 5 wells were prepared in each plate. Then, 100 μL of the ZnONP dispersed solution having a concentration of 300 μg mL^−1^ was kept in each well, followed by the incubation of the plates at 37 °C ± 0.1 °C for 24 h in a BOD incubator. The antibiotic penicillin-streptomycin (GIBCO, USA) was used as a positive control and distilled water was used as a negative control. The bacterial growth inhibition zone was measured after the incubation period. This study was repeated thrice, and the original results are shown as mean ± standard deviation.

#### 2.5.2. Cytotoxic application

##### 2.5.2.1. Cell culture and maintenance

HepG2 (human liver cancer cell line) and HaCat (human keratinocyte cell line) (procured from the National Centre for Cell Science; NCCS, Pune, India) were cultured and maintained in high glucose Dulbecco's modified Eagle medium (hDMEM; ThermoFisher Scientific, U.S.A.) supplemented with 10% (v/v) fetal bovine serum (FBS; ThermoFisher Scientific, U.S.A.) and 1% (v/v) streptomycin–penicillin and antimycotic solution (ThermoFisher Scientific, U.S.A.). Both types of cells were seeded separately in a T-25 tissue culture flask (NUNC, ThermoFisher Scientific, U.S.A.) and maintained at 37 °C with 5% CO_2_ in a humidified (85%) incubator (Esco Lifesciences, Singapore). The medium of each flask was replenished on every 48 h and cell passages were performed by subsequent expansion in a 1 : 3 ratio after attaining 70–80% confluency.

##### 2.5.2.2. Cytotoxicity assessment

The cytotoxicity of the synthesized nanoparticles (*e.g.*, GG-10, GG-20, and GG-40) was examined using MTT [3-(4,5-dimethylthiazol-2-yl)-2,5-diphenyltetrasodium bromide] (Sigma, U.S.A.) following the protocol described in a previously published article.^33^ In brief, cells (both HaCat and HepG2 cells in separate plates) at a density of 1 × 10^4^ cells per well were seeded in each well of a microtiter plate (96-well) and incubated at 37 °C with 5% CO_2_ for 24 h. Post incubation, the medium in each well was replenished with 180 μL of fresh medium and 20 μL of different concentrations of nanoparticles, *e.g.*, 50, 100, 250, 500, and 1000 μg mL^−1^ (prepared in fresh media), bringing the final concentration to 10 times that of the working concentration, which was 5, 10, 25, 50, and 100 μg mL^−1^, respectively. About 20 μL of medium without any nanoparticles (*i.e.*, 0 μg mL^−1^) was treated as the control. The plates were then incubated for 72 h at 37 °C with 5% CO_2_. After incubation, the spent medium was replenished with 180 μL of fresh medium and 20 μL of MTT solution (prepared in a concentration of 5 mg mL^−1^ in fresh media) was added to each well, followed by incubation at 37 °C with 5% CO_2_ for 4 h. Post incubation, the spent medium was removed and 200 μL of DMSO (Sigma, U.S.A.) was added to each well and incubated for 10 min in the dark to solubilize the formazan crystals. The absorbance was measured at 570 nm using a multiplate reader (Varioskan, ThermoFisher, U.S.A.).

#### 2.5.3. Molecular docking studies

**Fig. 1:**
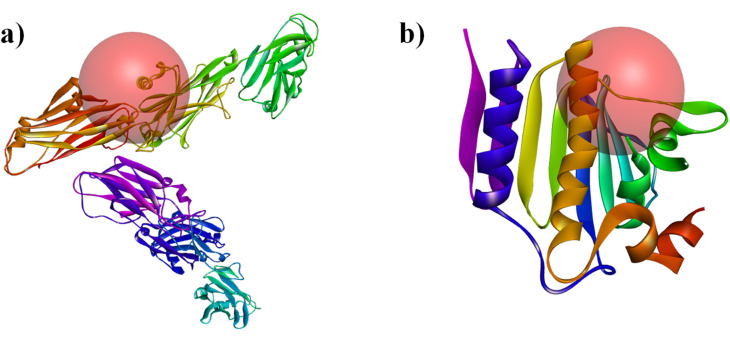
Targeted active binding site of receptors: (a) antigen-I/II carboxy-terminus (*S. mutans*, PDB id-3QE5) and (b) DNA gyrase B (*E. coli*, PDB id-6F86).

Molecular docking analysis was performed to understand the interaction between the coating phytoconstituents identified through GC-MS analysis and standard antibiotic (Penicillin G and streptomycin) (ligands) with the selected target protein *i.e.*, antigen-I/II carboxy-terminus (*S. mutans*, PDB ID: 3QE5) (Fig. 1a) and DNA gyrase B (*E. coli*, PDB ID: 6F86) (Fig. 1b) using AutoDock Tools 1.5.7.^34^ DNA gyrase B is an essential enzyme responsible for DNA replication in *E. coli* bacteria. Alternatively, antigen I/II carboxy-terminus, a protein found in *S. mutans*, facilitates colonization on the surface of teeth and promotes biofilm production, making it an important target in molecular docking.^35,36^ The structure of the selected receptors was retrieved from the Protein Data Bank site (https://www.rcsb.org/) in PDB format. Then, the protein was prepared in Biovia Discovery Studio software by deleting water molecules, defining and editing the binding site, adding polar H, *etc.* After that, Kollmann and Gasteiger charges were added (AutoDock Tools), and the file was saved in PDBQT format. The grid size was 15 × 15 × 15 Å points (*x*: 61.68, *y*: 28.33, *z*: 64.29) for 6F86 and 40 × 40 × 40 Å points (*x*: 72.27, *y*: 52.41, *z*: 126.18) for 3QE5. The 3D structure of all the ligands was downloaded from PubChem in SDF format, which was further converted into PDB format using Open Babel. AutoDock was used to prepare these ligands before they were used in the docking studies. Finally, the best binding pose (lowest binding energy) was visualized and analyzed using PyMoL. LigPlot+ software was used to see the interacting amin.

## 3. Results and discussion

### 3.1. Yield of extract, GC-MS analysis, and phytochemical identification

After the solvent had evaporated completely, the extracted residue weighed approximately 1.09 g, and the calculated yield was 10.9% (w/w). In the study conducted by Babich *et al.*, it was found that employing the Soxhlet extraction technique with methanol as the extractant (solvent), *G. glabra* extract produced the highest yield of approximately 21%.^37^

**Fig. 2:**
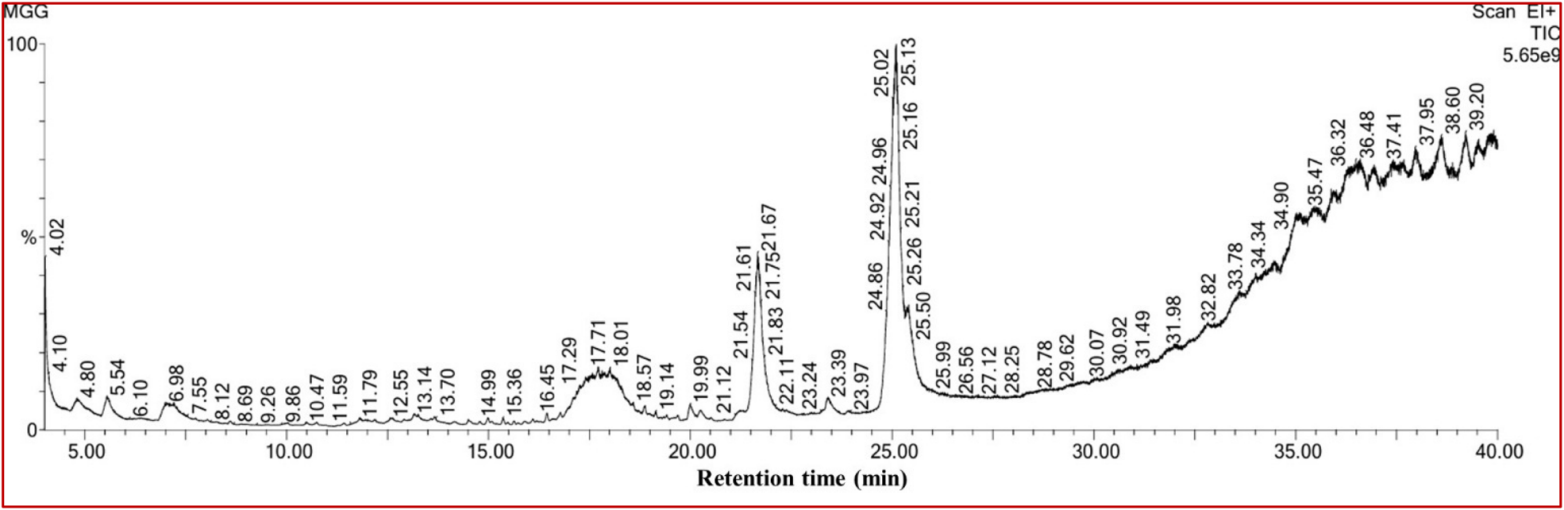
GC-MS chromatogram of the methanolic extract of the *G. glabra* root.

**Fig. 3:**
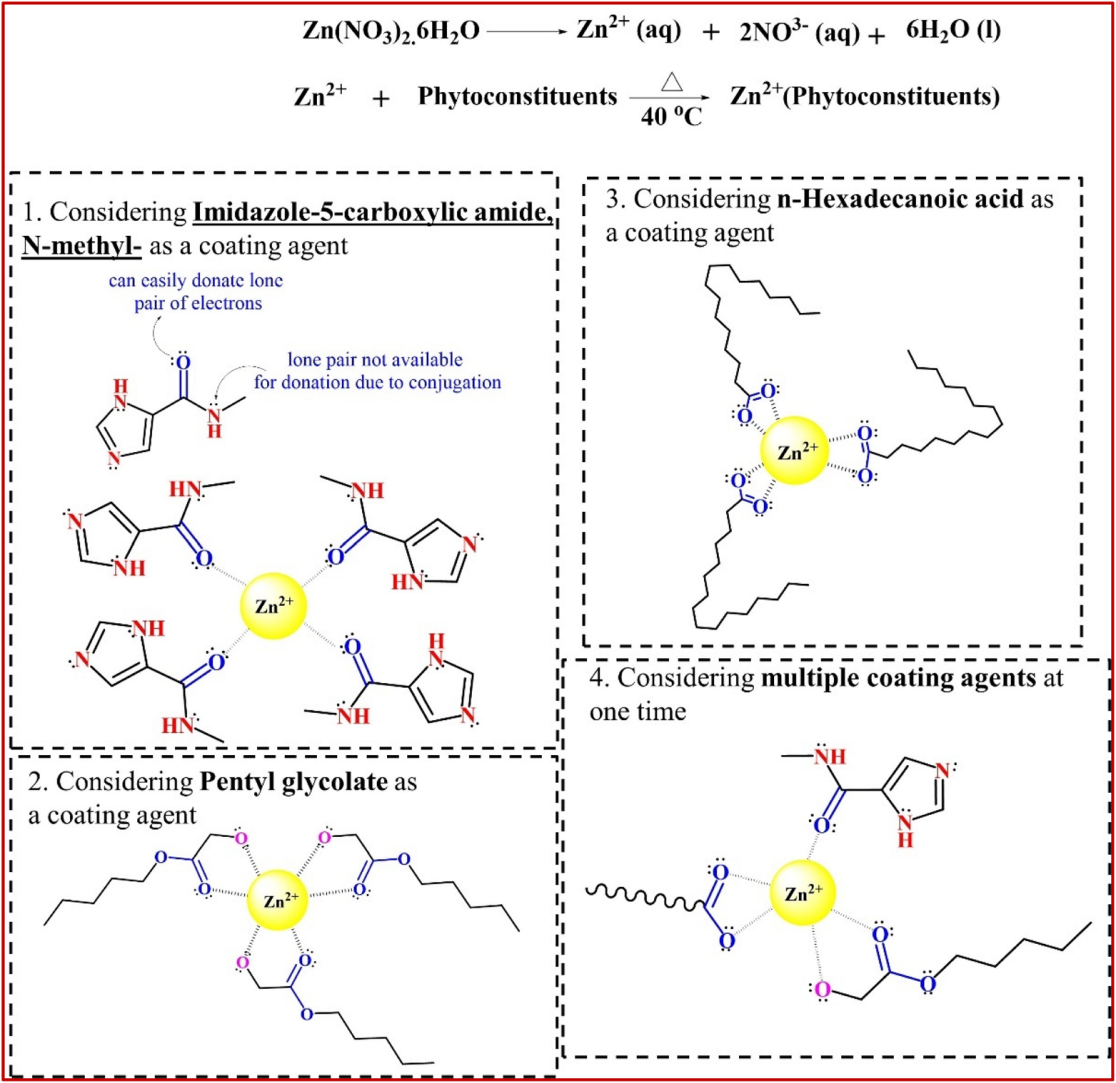
Probable mechanism for forming phytoconstituent-coated ZnO nanoparticles (considering some individual coating particles).

The GC-MS chromatogram of the *G. glabra* extract showed many peaks, as presented in Fig. 2, which indicated the presence of several phytoconstituents in the prepared extracts. However, the major identified bioactive compounds were *n*-hexadecanoic acid, pentyl glycolate, 9,12-octadecadienoic acid (*Z*,*Z*)-, *etc.*^38^ A list of the identified phytoconstituents with their retention time (RT), chemical structure, and peak area (%) is presented in SI Table S1. These phytochemicals may be the most likely ones to cap ZnO at the nanoscale, although more research is necessary to confirm this.^39^ The zinc ions present in the solution have great affinity towards these electron-rich phytoconstituents and they can form a layer by chelation or bridging mechanism (confirmed by FTIR spectra later in this paper).^40^ We proposed the probable interaction mechanism between zinc ions and phytoconstituents in Fig. 3. As shown in Table S1, 9,12-octadecadienoic acid (*Z*,*Z*)- and *n*-hexadecanoic acid were present abundantly with percentages of 35.23% and 11.41%, respectively. Thus, it could be illustrated that these two phytoconstituents are mainly responsible for the reduction and stabilization of ZnONPs. Subsequently, the coated phytochemicals can also significantly influence the potent antibacterial and cytotoxic activity of the samples. Similar to our study, four major chemical compounds were identified from the GC-MS chromatogram of *Aloe vera* leaf extract, which was used to synthesize ALE-ZnONPs.^41^

### 3.2. Ultraviolet-visible spectroscopy (UV-visible)

**Fig. 4:**
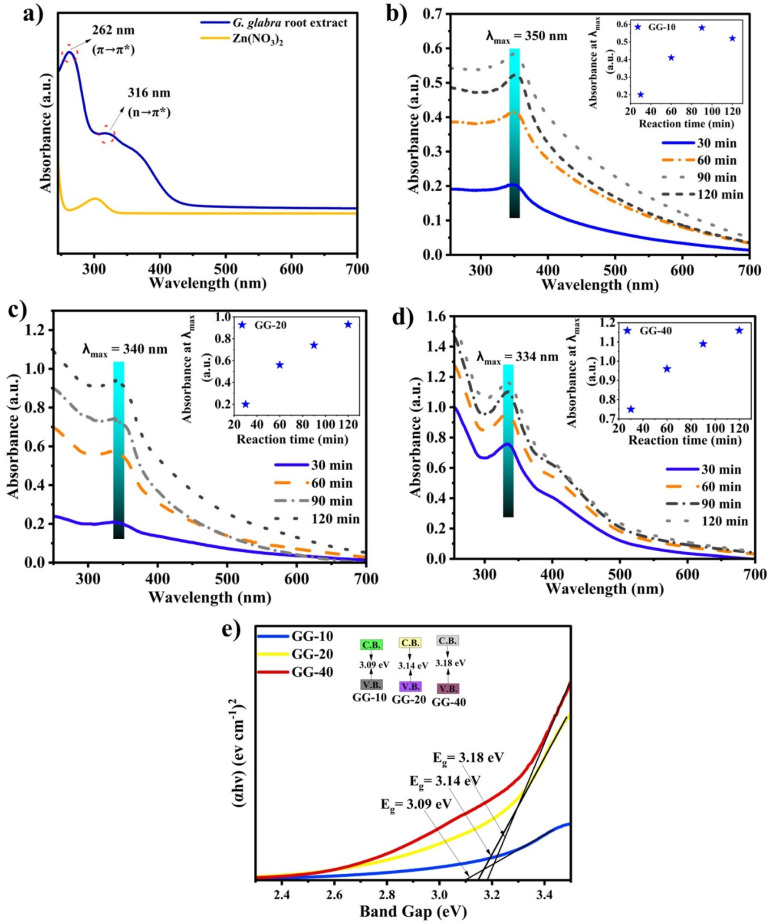
UV-visible spectra of (a) plant extract and the reaction time-dependent absorption spectra of (b) GG-10 (10 mL extract), (c) GG-20 (20 mL extract), and (d) GG-40 (40 mL extract) samples. (e) Band gap energy of three samples.

UV-visible spectroscopy is the preferred technique for the initial characterization of metallic nanoparticles. Due to surface plasmon resonance (SPR) phenomena (matching the collective oscillation of surface electrons with the specific frequency of incident light produces resonance, leading to the maximum absorbance), metallic nanoparticles commonly show absorption spectra in the UV-visible region (200–800 nm).^42^ As presented in Fig. 4, the effect of plant extract volumes was assessed by varying the ratio of plant extract volume to the precursor (Zn(NO_3_)_2_·6H_2_O), while keeping all other reaction parameters constant (*i.e.*, temperature ∼40 °C, pH ∼9.0, and rotation speed ∼700 rpm). The green color of the plant extract showed two characteristic UV-visible peaks at ∼262 nm and ∼316 nm (as shown in Fig. 4a) possibly due to π → π* and n → π* electronic transitions, respectively.^43,44^ These two transitions may be associated with the electronic transitions of the keto group (–C=O) present in the phytochemical, which was further confirmed by FTIR and GC-MS. For differentiation, we recorded the maximum absorbance peak of the precursor (Zn(NO_3_)_2_) (301 nm), as illustrated in Fig. 4a, along with the plant extract. Alternatively, the extract-mediated as-synthesized ZnONPs showed the characteristic SPRs peak at ∼350, ∼340, and ∼334 nm for the GG-10 (Fig. 4b), GG-20 (Fig. 4c), and GG-40 (Fig. 4d) samples with the corresponding band gap (*E*_g_) (Fig. 4e) of 3.09, 3.14 and 3.18 eV, respectively.^45^ Furthermore, the broadness of the absorbance peak was the lowest using a large amount (40 mL) of reducing/stabilizing agent (*i.e.*, plant extract), indicating the formation of less agglomerated particles.^46^ The *E*_g_ values were determined by extrapolating the Tauc plot to the *x*-axis. Here, the band gap increased with an increase in the volume of extract, indicating the formation of smaller particles. The strong absorption at the UV region (334–350 nm) could be due to the electron transfer from the valence band (V.B.) to the conductance band (C.B.), *i.e.*, O2p-Zn3d.^47^ In addition, we also observed a blue shift or hypsochromic shift, indicating the formation of smaller-sized nanoparticles.^48^ However, the chemically synthesized ZnONPs (Chem.-ZnO) absorbed at the longest wavelength among the samples, which was about 354 nm, and the band gap for Chem.-ZnO was 3.06 eV, as shown in Fig. S2. Therefore, Chem.-ZnO had the largest particle size, and this was well supported by Darvishi *et al.*^49^ During the time optimisation, the highest intensity of the absorbance peak was found after 90 min for 10 mL, while 120 min was the ideal time for large volumes, *i.e.* 20 and 40 mL of extract (see SI Table S2 for the corresponding absorbance values at different reaction times). This observation implies that a large quantity of plant extract requires extra time to complete the reaction. Time-dependent absorbance values were also observed in a previous report.^50,51^ Tilahun *et al.* optimized different reaction conditions such as temperature, time, and concentration of Zn(CH_3_COO)_2_ using response surface methodology (RSM) and found that the optimal reaction time was ∼95 min.^32^ In a recent study by Ramya *et al.*, the formation of ZnONPs was confirmed using UV-visible spectroscopy, with an absorption band observed at ∼345 nm.^20^ Similar results were also observed in another study by Rajendran *et al.*^52^ However, *Glycyrrhiza glabra* seed aqueous extract and *Cinnamomum verum*-mediated ZnONPs showed an absorption peak at relatively lower and higher wavelengths of 275 nm (ref. 51) and ∼370 to 375 nm,^53^ respectively.

### 3.3. XRD spectroscopy

**Fig. 5:**
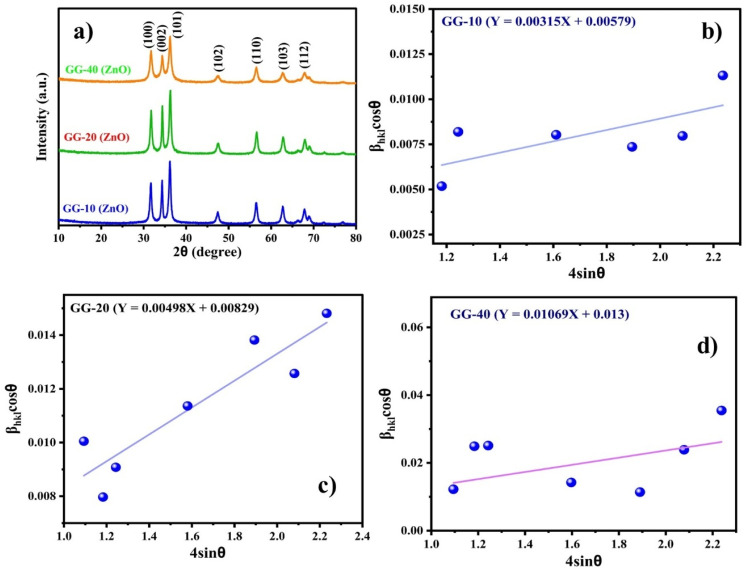
(a) XRD pattern of all three samples. Williamson–Hall plot of (b) GG-10, (c) GG-20, and (d) GG-40.

**Fig. 6:**
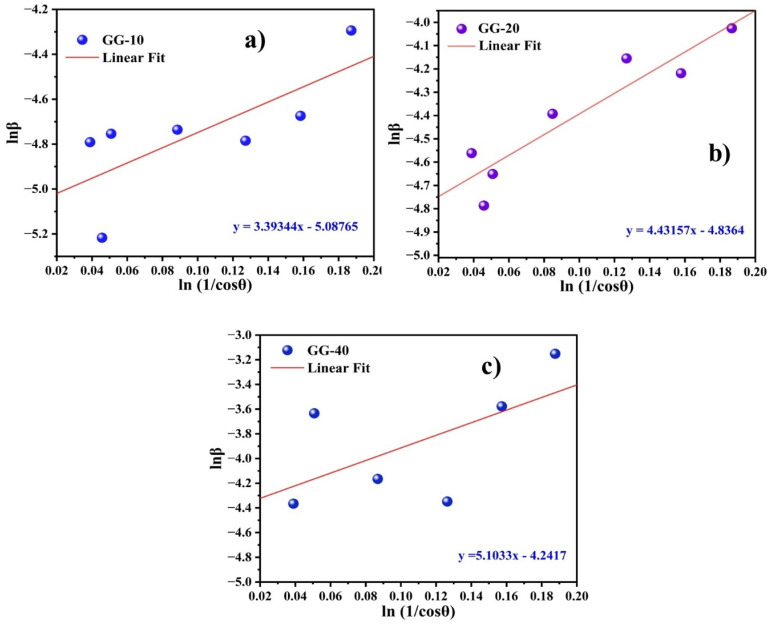
Modified Scherrer plot of (a) GG-10, (b) GG-20, and (c) GG-40.

Powder XRD crystallography revealed the hexagonal crystalline structure and high purity of the as-synthesized ZnONPs, as shown in Fig. 5a. Seven major diffraction peaks were observed for the GG-10 sample, at 31.7°, 34.3°, 36.2°, 47.5°, 56.5°, 62.8° and 67.9°, which correspond to the (100), (002), (101), (102), (110), (103) and (112) Miller indices, respectively. These peaks are well accredited by the standard JCPDS XRD pattern of the ZnONPs (JCPDS file number: 800075). A similar pattern was observed for the other two samples (GG-20 and GG-40). The average crystallite size (*D*) of the as-synthesized ZnONPs was calculated using the Scherrer equation (see SI Table S3) and was determined to be 18.92, 12.74, and 10.17 nm for GG-10, GG-20, and GG-40, respectively, whereas a similar decreasing trend for the same was found by the W–H method from 25.01 to 11.14 nm, as shown in Table 1. Here, the crystal lattice strain may be responsible for the slightly higher crystallite size value obtained from the W–H plot.^54^ In addition, the microstrain contribution (*ε*) was calculated from the slope of the W–H plot for the three ZnONP samples (as shown in Fig. 5b–d), respectively, and the estimated values are presented in Table 1.^55^ More or less similar crystallite size values were also obtained using the modified Scherrer plot for GG-10 (Fig. 6a), GG-20 (Fig. 6b), and GG-40 (Fig. 6c). The details including 2*θ*, FWHM, *etc.* for the calculation of *D*, using the Debye–Scherrer, W–H plot, and modified Scherrer plot methods are presented in SI Tables S3–S5, respectively. The decreasing order of crystallite size of ZnONPs observed might be due to the presence of a large number of capping agents (phytoconstituents) on the nanoparticle surface, preventing the aggregation of the molecules.^56^ The lattice constants (*a* and *c*) were also determined using the following equation:6
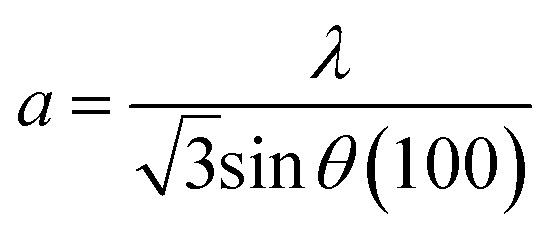
and7
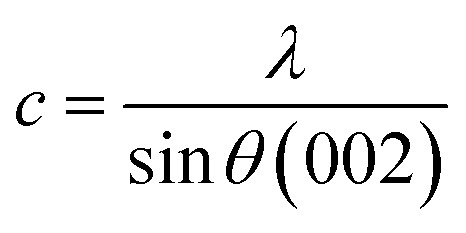


**Table 1:** Variation in the average crystallite size (*D*), lattice constant (*a* and *c*), and lattice strain (*ε*) with extract volume

Sample	Average crystallite size (in nm)			Crystal strain (*ε*) from W–H plot (×10^−3^)	Lattice constant (Å)		*c*/*a*
	Debye–Scherrer method	Williamson–Hall method	Modified Scherrer method		*a* = *b*	*c*	
GG-10	18.92	25.01	23.46	3.15	3.2511	5.2106	1.6027
GG-20	12.74	17.47	18.25	4.98	3.2516	5.2050	1.6007
GG-40	10.17	11.14	12.08	10.69	3.2496	5.2040	1.6014

These values were almost similar for all three samples (as presented in Table 1), which suggests their closeness to the core ZnO crystal structure. In an early study by Dayakar *et al.*, the crystallite size of ZnONPs (determined by Scherrer and W–H plot) decreased when different concentrations (5–20 mL) of *Ocimum tenuiflorum* extract were used.^57^

### 3.4. FTIR spectroscopy

**Fig. 7:**
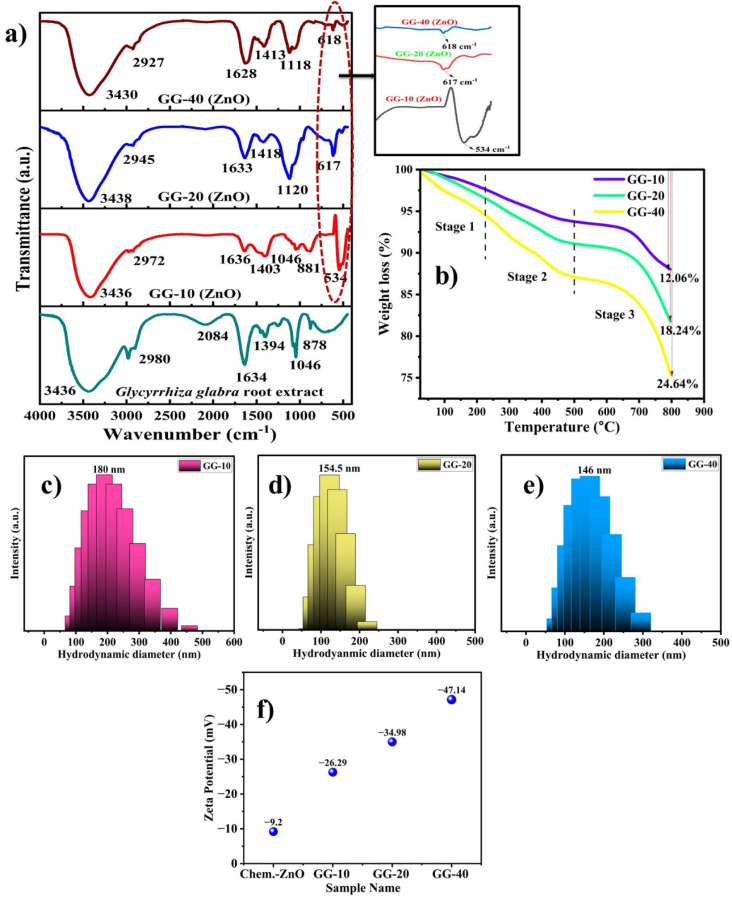
(a) FTIR spectrum of *G. glabra* extract and the as-synthesized ZnONPs in the spectral window of 450–4000 cm^−1^, (b) TGA trace of all three ZnO samples, DLS analysis of (c) GG-10, (d) GG-20, and (e) GG-40, and (f) zeta potential analysis of Chem.-ZnO (chemically synthesized ZnONPs) and all three phytoconstituent-coated ZnONPs (biosynthesized ZnONPs).

Identification of different functional groups on the surface was done by FTIR spectroscopy.^58^Fig. 7a shows the FTIR spectrum of *G. glabra* extract alone and all the nanoparticle samples. The strong and broad peak at 3418–38 cm^−1^ corresponds to the O–H stretching vibration due to the presence of water molecules or polyphenolic molecules. The stretching vibration modes of C–H were observed at 2972, 2945, and 2927 cm^−1^.^59^ The peaks at 1636, 1633, and 1628 cm^−1^ were found due to the C=O stretching of amide or carboxylic acid groups.^60^ Similarly, the bands at 1403–1418, 1046–1120, and 881 cm^−1^ are referred to as O–H bending, C–O stretching, and C=C bending vibration, respectively. The peaks at 534, 617, and 618 cm^−1^ are the characteristic absorption of the Zn–O bond (zinc and oxygen bond stretching). In all cases, a slightly different wavenumber value was observed in the FTIR spectrum, which may be due to the variation in the coating environment on the particle surface.^61^ The FTIR spectrum analysis of both the *G. glabra* extract and nanoparticle sample confirmed the existence of an organic molecular layer on the nanoparticle surface. This indicates that several organic compounds such as polyhydroxy groups, carboxylic acids, and esters present in the as-prepared extract are involved in the reduction process, and also contribute to stabilizing the nanoparticles by forming a protective layer around them.^62^ Our FTIR result was also aligned with the compound identification from GC-MS analysis.

### 3.5. Thermogravimetric analysis (TGA)

TGA was conducted to study the thermal properties of ZnONPs and confirm the presence of phytoconstituents as capping agents on their surface.^39^ The recorded thermograms for all three samples and plant extract (*G. glabra*) alone are shown in Fig. 7b and SI Fig. S3, respectively. The ZnONP sample shows a three-step thermal decomposition at 25–225 °C, 225–502 °C, 502–800 °C, and the possible decomposed substances are water molecules (loosely bound) or any volatile phytoconstituents, physically or chemically absorbed organic coatings (strongly bound) and complete decomposition of organic moieties from the surface of the nanoparticles. A total weight loss of 12.06% up to 800 °C was observed for the GG-10 sample, whereas 18.24% and 24.64% weight loss were observed for the GG-20 and GG-40 samples, respectively (Table S6 in the SI file for stepwise weight reduction). This weight loss trend suggests that the nanoparticle samples had an increased coating. However, *G. glabra* extract alone showed a total weight loss of about 31.52% up to 500 °C due to the evaporation of water molecules and decomposition of high concentrations of organic moieties (*i.e.*, phytoconstituents). According to the early research by Chunduri *et al.*, uncoated ZnONPs only lose 4.2% of their initial weight when heated to 800 °C.^63^ Our TGA observation was compared with the earlier published result (see SI Table S7).

### 3.6. DLS analysis

The hydrodynamic size (*z*-average) and size distribution of the as-synthesized samples were investigated using the dynamic light scattering technique, as presented in Fig. 7c–e. The *z*-average for the GG-10 (Fig. 7c), GG-20 (Fig. 7d), and GG-40 (Fig. 7e) samples were found to be ∼180, ∼154, and ∼146 nm, while their polydispersity index (PDI) values were 0.245, 0.348, and 0.348, respectively. The observed PDI values indicated the formation of a moderate to broad size distribution of particles.^64^ However, a bigger particle size value was recorded due to the aggregation of the synthesized ZnONPs in the dispersion medium. Our result is consistent with the already published report on biogenic ZnONPs.^65^

### 3.7. Zeta potential analysis

The colloidal stability of the as-synthesized phytoconstituent-coated ZnONPs was measured by recording the magnitude of *ζ*-potential in millivolts (mV), which was also compared with the chemically synthesized ZnONPs. The net electrical charge of the nanoparticles in solution can generally be precisely measured using the *ζ*-potential, which indicates the probable long-term stability of colloidal solutions.^66^ The average *ζ*-potential value increased from −26.29 mV (for GG-10) (Fig. S4a) and −34.98 mV (for GG-20) (Fig. S4b) to −47.14 mV (for GG-40) (Fig. S4c); however, a small *ζ*-potential value (−9.2 mV) was observed in the case of chemically synthesized ZnONPs (Chem.-ZnO) (Fig. S4d). A colloidal solution with a *ζ*-potential above ±15 mV is considered to have good stability.^17^ In this study, the observed incremental *ζ*-potential indicates the greater stability of the samples in a common dispersion medium (aqueous), as shown in Fig. 7f. The presence of diverse phytochemicals on the surface of ZnONPs prevents their agglomeration because of their strong electrostatic repulsion, and thereby results in a higher negative surface charge for the phytoconstituent-coated ZnONPs.^66^ Conversely, the chemically synthesized ZnONPs exhibited the lowest zeta potential, probably as a result of the absence of stabilizing agents that were otherwise present in the samples coated with phytochemicals. The outcome of this study was also in good agreement with a previous study on ZnONPs synthesized using *A. indica* leaf extract.^67^ The UV-visible, XRD, DLS, and zeta potential results revealed that the particle size, size distribution, and other physical properties of the nanoparticles can be influenced by the quantity of plant extract.

### 3.8. FESEM, EDX, and elemental mapping analysis

**Fig. 8:**
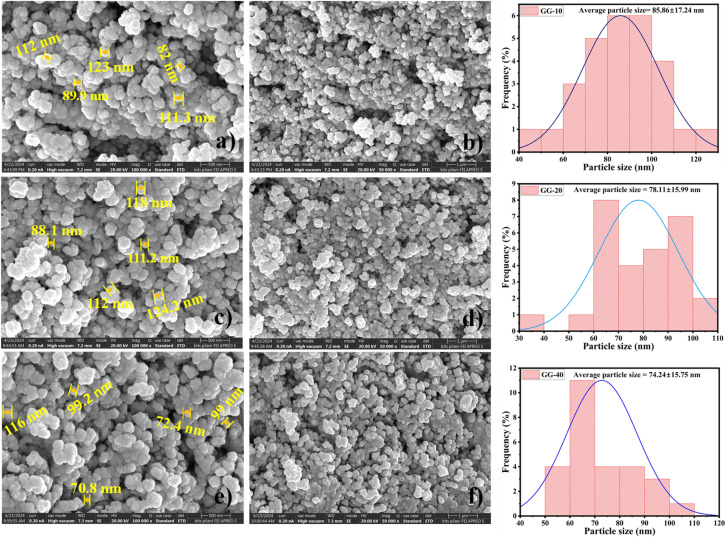
FESEM images of GG-10 (a and b), GG-20 (c and d), and GG-40 (e and f) samples at two different magnifications with corresponding histograms.

**Fig. 9:**
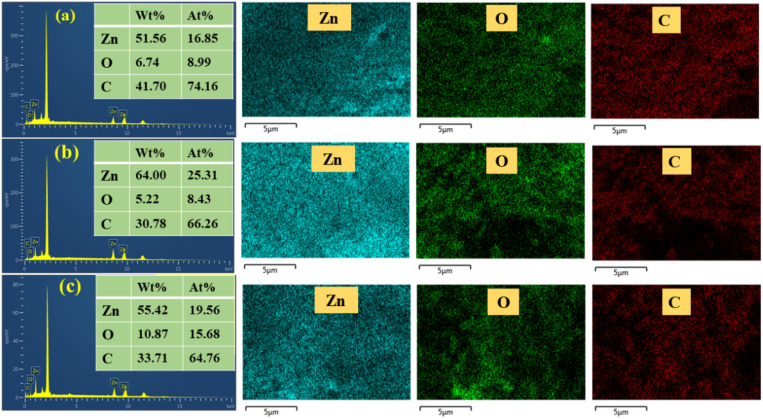
EDX spectrum of the GG-10 (a), GG-20 (b), and GG-40 (c) samples with elemental mapping of zinc, oxygen, and carbon.

Fig. 8 displays the FESEM images of all three samples with a histogram showing the average particle size. This result revealed a roughly spherical morphology with some aggregation. However, the particle size was approx. 85 nm, 78 nm, and 74 nm (determined using ImageJ software) for the GG-10, GG-20, and GG-40 samples, respectively. This suggests that a high amount of plant extract can synthesize comparatively smaller-sized nanoparticles. However, the difference in particle size among the three as-synthesized ZnONPs was not very significant, which was also previously explained in the XRD and DLS analysis. Our findings were also consistent with the study conducted by Faisal *et al.*, with a size in the range of 43.3–83.1 nm.^17^ The EDX patterns of all the prepared samples of ZnONPs (shown in Fig. 8) show the existence of Zn, O, and C elements. The appearance of carbon might be due to the outer organic layer on the particle surface. The EDX pattern also indicates the elevated purity of all three samples. In the GG-10 samples, the atomic % of oxygen, carbon, and zinc were 8.99, 74.16, and 16.85, respectively. Alternatively, the weight % of oxygen, carbon, and zinc was 6.74 ± 0.24, 41.70 ± 0.44, and 51.56 ± 0.41, respectively. However, both the weight % and atomic % varied in the case of the other two samples, *i.e.* GG-20 and GG-40 (inserted in Fig. 8). According to the elemental mapping, the nanoparticle surface exhibits a homogeneous distribution of all the constituent elements (Fig. 9).

### 3.9. Antibacterial activity of as-synthesized ZnONPs

**Fig. 10:**
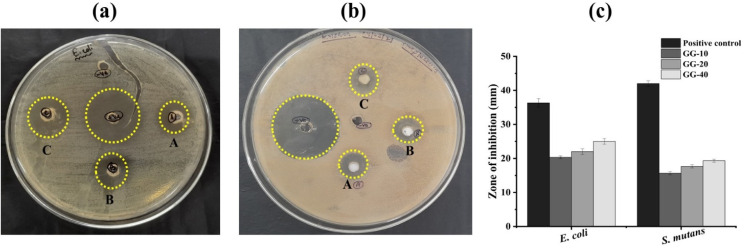
Agar well diffusion assay against (a) *E. coli* and (b) *S. mutans*, where wells A = GG-10, B = GG-20, C = GG-40, +ve = standard antibiotic, and −ve = *d*H_2_O. (c) Bar graph showing comparative antibacterial activity. Note: *A minor crack is visible in panel A, E. coli, which was caused during handling. This did not affect the bacterial growth and the measurement of the zone of inhibition.*

The agar well diffusion assay showed that GG-40 (ZOI = 25 ± 0.81 mm) exhibited the maximum antibacterial activity against *E. coli* compared to GG-10 (ZOI = 20.3 ± 0.40 mm) and GG-20 sample (ZOI = 22 ± 0.81 mm) (Fig. 10a). Again, these samples showed slightly lower antibacterial activity against *S. mutans* with an inhibition zone of 15.66 ± 0.47, 17.66 ± 0.47 and 19.33 ± 0.47 mm for GG-10, GG-20, and GG-40 ZnONPs, respectively (Fig. 10b). However, the standard antibiotic (used as positive control) possesses higher antibacterial activity than all three samples of ZnONPs with an inhibition zone of 36.33 ± 1.24 and 42 ± 0.81 mm against *E. coli* and *S. mutans*, respectively. A bar graph was plotted in Fig. 10c to understand the antibacterial properties of all treatments. These research findings also suggested that the antibacterial properties of ZnONPs can be improved by increasing the volume of plant extract used in their production. It is generally agreed that higher concentrations of plant extracts produce more toxic and hydrophilic metal nanoparticles.^68^ The increased hydrophilicity of the nanoparticles enhances their antibacterial activity by accumulating a large number of particles on the bacterial surface. The generation of reactive oxygen species (ROS) such as hydrogen peroxide (H_2_O_2_), hydroxyl radical (HO˙^−^), and superoxide anions (O_2_˙^−^) is one of the primary antibacterial mechanisms of ZnONPs, which are responsible for the disruption of the cell membrane, DNA damage and killing of bacteria.^69^ In an early study, the ability of ZnONPs synthesized using *A. indica* leaf extract to produce ROS in different bacterial cells was confirmed by employing the DCFDA assay.^67^ Ahmed *et al.*^70^ in another work, through the NBT assay, confirmed that ZnONPs can produce O_2_˙^−^ in a dose-related manner. Additionally, the coated phytoconstituents can induce the generation of more ROS. Furthermore, similar to our observation, many previous studies also claimed that ZnONPs exhibit greater antibacterial activity against Gram-negative bacterial strains compared to Gram-positive strains.^4,71^ This might be due to the difference in their cell wall structure. Mainly, Gram-positive bacteria have a thicker outer peptidoglycan layer than Gram-negative bacteria, which protects them from the action of antimicrobial agents.^72^

### 3.10. *In vitro* cytotoxic activity of as-synthesized ZnONPs

**Fig. 11:**
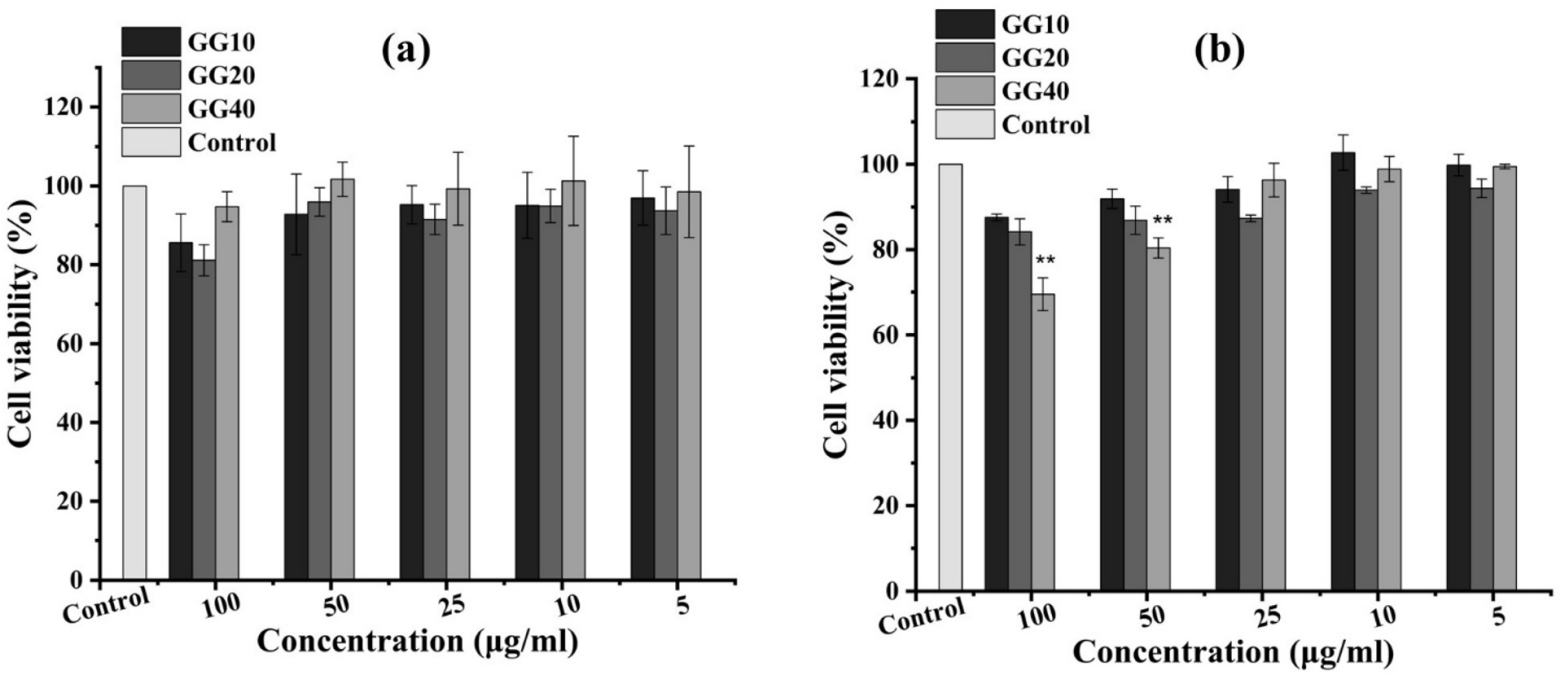
Cytotoxicity assay using different concentrations of nanoparticles on HaCat (a) and HepG2 cell lines (b). Values are presented as mean ± standard deviation, *n* = 3, where **p ≤ 0.01.

In this study, the cytotoxicity of the samples was evaluated using the MTT reduction assay, wherein yellow tetrazolium dye was reduced to purple formazan granules by mainly mitochondrial succinic dehydrogenases of metabolically active cells, although few cytosolic enzymes such as nicotinamide adenine dinucleotide (NAD) H-dependent oxidoreductase and flavin oxidase might also be involved.^73^ The rate of this conversion of tetrazolium to formazan was directly linked to the metabolic activity of the cells tested and the number of mitochondria present in the cells.^74^ This study demonstrated that the nanoparticles responded to the dose, synthesis procedures, and cell types. For instance, GG-10, GG-20, and GG-40 did not show significant differences in cell viability up to 50 μg per mL concentration when tested against normal keratinocyte human (HaCaT) cells, as presented in Fig. 11a, whereas GG-40 supported (69.5 ± 3.8)% and (80.3 ± 2.3)% cellular viability at the concentration of 100 and 50 μg mL^−1^, respectively, when tested against human liver cancer (HepG2) cells (*p* ≤ 0.01), as shown in Fig. 11b. Nearly 100% cell viability was observed at a concentration up to ∼50 μg mL^−1^ against HaCaT cells for all three samples, which indicated their non-toxic nature towards normal cells. Additionally, the cytotoxicity results showed that the percentage of cell viability decreased with an increase in concentration from 5 to 100 μg mL^−1^ for all three ZnONPs against HepG2 cancer cells. Some previous studies also demonstrated that plant-based ZnONPs have dose-dependent cytotoxicity activity.^30,75,76^ A detailed comparison study with previously reported work is presented in Table 2 to showcase the novelty of the *G. glabra* root extract-mediated ZnONPs. In the previous study by Meiguang Zheng *et al.*, *G. glabra* seed aqueous extract-mediated ZnONPs showed a dose-dependent cytotoxic effect against human U-87 glioblastoma cells with IC_50_ of 30 μg L^−1^.^51^ According to the research by Vijyakumar *et al.*, Ln-ZnONPs had a concentration-dependent harmful effect on A549 (human lung cancer epithelium) cancer cells but were non-toxic to normal murine macrophage RAW264.7 cells. They further confirmed these results by visualizing under a phase contrast microscope.^77^ The present findings conclusively demonstrate that the quantity of plant extract (in this case, *G. glabra* root extract) used as a capping agent possibly affects the cytotoxic activities exhibited by the synthesized ZnONPs (GG-10, GG-20, and GG-40).

**Table 2:** A comparison study of the cytotoxic activity of ZnONPs synthesized through chemical and biological methods

Method of nanoparticle synthesis	Size	Cell types	Assay	Results	Reference
Biological (*Deverra tortuosa* aerial part)	9.26–31.18 nm	Human lung epithelial carcinoma (A549), normal lung fibroblast (WI38), and colorectal epithelial adenocarcinoma (Caco-2)	MTT	The IC_50_ values of ZnONPs were ∼83, ∼50, and ∼434 μg mL^−1^ for A549, Caco-2, and WI38 cells, respectively	78
Chemical (sol–gel)	∼7 nm	Cervical cancer (HeLa) and normal fibroblasts (MSU1.1)	WST-1	Normal and cancer cells show ∼80% cell viability at low concentrations (10 μg mL^−1^), while it diminishes up to ∼40% at 100 μg mL^−1^ concentration	79
Biological (*Artocarpus heterophyllus* leaf)	12–24 nm	Normal vero and colon cancer HCT-116 cell line	MTT	The sample showed high toxicity against both normal as well as cancer cell lines with IC_50_ ∼30 and 20 μg mL^−1^, respectively	76
Biological (*Phlomis* leaf)	∼79 nm	Normal fibroblast L929	MTT	∼80% cell viability at 90 μg per mL concentration	80
Biological (*Punica granatum* fruit)	∼32 and 82 nm at 600 °C and 700 °C annealing temperatures, respectively	Colon normal (CCD112) and cancer cells (HCT116)	Cell proliferation	Samples showed similar cytotoxicity against normal and human cells with IC_50_ value ∼25 μg mL^−1^	81
Chemical (precipitation method)	15–20 nm	Normal mouse fibroblast NIH 3T3	MTT	∼65% and ∼78% cell viability were observed for ZnO and modified ZnO against tested cells, respectively	82
Biological (*G. glabra* root)	74–85 nm	Normal human keratinocyte (HaCaT), and liver cancer cells (HepG2)	MTT	All three samples of ZnONPs showed ∼85% cell viability at 100 μg per mL concentration against normal cells. Whereas, the GG-40 sample showed 69.5% ± 3.8% cell viability at 100 μg mL^−1^ against cancer cells	This study

### 3.11. Molecular docking studies

**Fig. 12:**
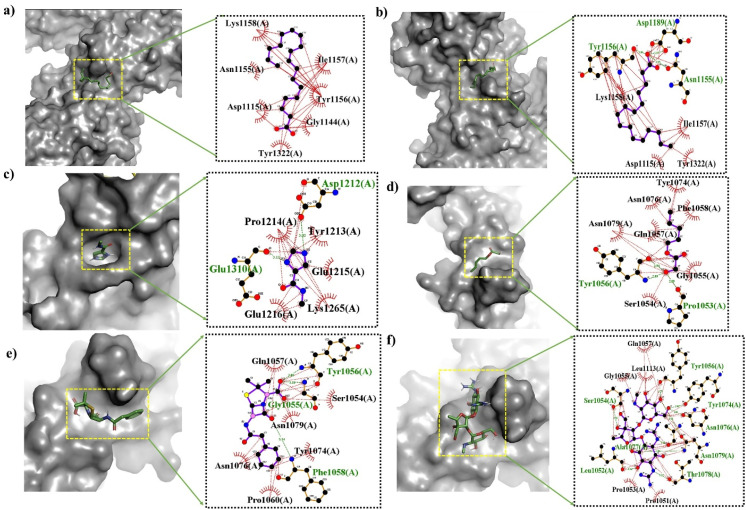
3D (left) and 2D (right) representations of the molecular docking interactions of (a) 9,12-octadecadienoic acid (*Z*,*Z*)-, (b) *n*-hexadecanoic acid, (c) imidazole-5-carboxylic amide, *N*-methyl-, (d) pentyl glycolate, (e) penicillin G (standard), and (f) streptomycin (standard) against antigen-I/II (*S. mutans*, PDB id-3QE5) proteins.

**Fig. 13:**
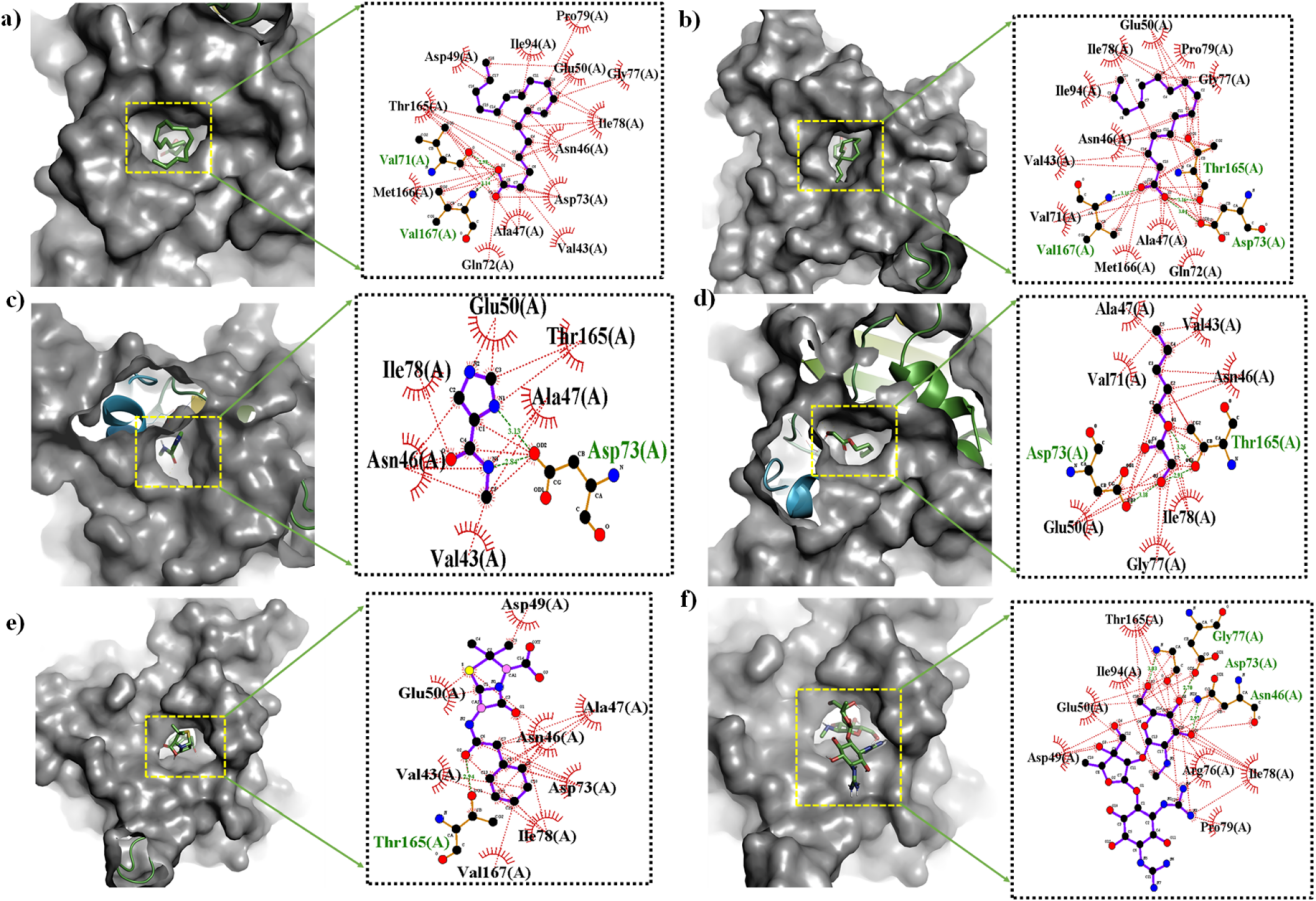
3D (left) and 2D (right) representations of the molecular docking interactions of (a) 9,12-octadecadienoic acid (*Z*,*Z*)-, (b) *n*-hexadecanoic acid, (c) imidazole-5-carboxylic amide, *N*-methyl-, (d) pentyl glycolate, (e) penicillin G (standard), and (f) streptomycin (standard) against DNA gyrase B (*E. coli*, PDB id-6F86) proteins.

Given that the phytoconstituent-coated ZnONPs demonstrated excellent bactericidal efficacy *in vitro*, *in silico* molecular docking investigations were conducted to determine the likely mechanism of interaction between the phytoconstituents (ligand) and target proteins (receptor) in *S. mutans* and *E. coli*.^36^ A higher negative value in molecular docking denotes better receptor binding affinity. All four phytoconstituents, *i.e.*, 9,12-octadecadienoic acid (*Z*,*Z*)-, *n*-hexadecanoic acid, imidazole-5-carboxylic amide, *N*-methyl-, and pentyl glycolate, showed significant binding affinity, as shown in Table 3. Table 3 also presents the interaction mechanisms (*i.e.*, H-bonding and hydrophobic) of all the selected ligands with surrounding amino acids. However, among the phytoconstituents, 9,12-octadecadienoic acid (*Z*,*Z*)-, and *n*-hexadecanoic acid exhibited comparatively higher docking scores of −5.5 kcal mol^−1^ each with DNA gyrase B in *E. coli*, while the docking score was −4.8, and −4.5 kcal mol^−1^ with antigens-I/II in *S. mutans*, respectively. Furthermore, *in silico* results revealed that the phytoconstituents have greater affinity toward Gram-negative *E. coli* than Gram-positive *S. mutans*, which supports our *in vitro* outcomes. 9,12-Octadecadienoic acid (*Z*,*Z*)- had hydrophobic interactions with many of the amino acid residues in *S. mutans* such as Lys1158, Asn1155, Asp1115, and Tyr1332 (Fig. 13a), and additionally, it showed hydrophobic interactions with Asp49, Thr165, Met166, Gln72, Ala47, *etc.*, and had strong H-bonding with Val67 and Val71 in the *E. coli* target (Fig. 13a). These interactions with amino acid residues are essential for destroying the protein structure and obstructing the capacity to form biofilms. The 3D and 2D representations of the docking outcomes for all the considered ligands with *S. mutans* and *E. coli* are shown in Fig. 12b–e and 13b–e, respectively. Each phytoconstituent had a unique binding mechanism and different binding energy with the target receptors, which is caused by their structural diversity. According to the results of the molecular docking, ZnONPs coated with phytoconstituents demonstrated distinct antibacterial strategies by impairing the function of important proteins, rupturing the membrane integrity, *etc.*^83,84^

**Table 3:** *In silico* molecular docking results of phytoconstituents identified in the *G. glabra* root by GCMS analysis and standard antibiotic with proteins (*i.e.*, DNA gyrase B in *E. coli* and antigen-I/II carboxy-terminus in *S. mutans*)

S/no.	Ligand name	PubChem Id	3QE5 (*S. mutans*)			6F86 (*E. coli*)		
			Binding energy (kcal mol^−1^)	Protein–ligand complex interaction details		Binding energy (kcal mol^−1^)	Protein–ligand complex interaction details	
				H-bond interactions	Hydrophobic interactions		H-bond interactions	Hydrophobic interactions
1	9,12-Octadecadienoic acid (*Z*,*Z*)-	5280450	−4.8	—	Lys1158, Asn1155, Asp1115, Tyr1332, Gly1144, Tyr1156, Ile1157	−5.5	Val67, Val71	Asp49, Thr165, Met166, Gln72, Ala47, Asp73, Asn46, Ile78, Gly77, Glu50, Pro79, Ile94
2	*n*-Hexadecanoic acid	985	−4.5	Tyr1156, Asp1189, Asn1156	Lys1158, Asp1115, Tyr1322, Ile1157	−5.5	Thr165, Asp73, Val167	Ile78, Ile94, Val43, val71, Met166, Ala47, Gln72, Gly77, Pro79, Glu50, Asn46
3	Imidazole-5-carboxylic amide, *N*-methyl-	565659	−4.6	Glu1310, Asp1212	Pro1214, Tyr1213, Lys1265, Glu1216	−5.2	Asp73	Glu50, Ile78, Asn46, Val43, Ala47, Thr165
4	Pentyl glycolate	225038	−4.0	Pro1053, Tyr1056	Asn1079, Asn1076, Gln1057, Phe1058, Gly1055, Ser1054	−4.9	Thr165, Asp73	Ala47, Val71, Glu50, Gly77, Ile78, Asn46,Val43
5	Streptomycin (standard)	19649	−6.0	Ser1054, Leu1052, Thr1078, Asn1079, Asn1076, Tyr1074, Tyr1056	Gln1057, Gly1055, Leu1113, Pro1053, Pro1051	−6.0	Gly77, Asp73, Asn46	Thr165, Ile94, Glu50, Asp49, Pro79, Arg76, Ile78
6	Penicillin G (standard)	5904	−6.1	Gly1055, Tyr1056, Phe1058	Gln1057, Asn1076, Pro1060, Tyr1074, Asn1079, Ser1054	−6.1	Thr165	Glu50, Val43, Val167, Ile78, Asp73, Asn46, Ala47, Asp49

## 4. Conclusion

The outcome demonstrates a facile and eco-friendly approach for the production of biocompatible ZnONPs using *G. glabra* root extract. Several secondary metabolites (phytochemicals) such as *n*-hexadecanoic acid, pentyl glycolate, and 9,12-octadecadienoic acid (*Z*,*Z*)- were mainly responsible for the formation of phytochemical-coated ZnONPs, as evident by the GC-MS analysis. This study showed that many of the physicochemical properties of ZnONPs, such as band gap, crystallite size, hydrodynamic size, thermal behaviour, and stability, were significantly affected by the amount of *G. glabra* extract. The antibacterial application of ZnONPs suggests that ZnONPs from 40 mL plant extract have better bacterial inhibitory potential against *E. coli* (25 ± 0.81 mm) and *S. mutans* (19.33 ± 0.47 mm) in comparison to that from 10-, and 20-mL extract, which was well supported by our *in silico* studies. Molecular docking confirms there was a strong interaction (−4.0 to −5.5 kcal mol^−1^) between the bacterial proteins and phytochemicals. Furthermore, the as-synthesized ZnONPs showed no toxicity (cell viability nearly 100%) against the normal keratinocyte cell line (HaCaT) up to a concentration of 50 μg mL^−1^, depicting the biocompatible nature of the samples. However, the samples showed comparatively high toxicity against HepG2 cancer cells (cell viability of approx. 69%). Thus, these findings suggest that the active phytoconstituents in the mulethi extract can produce biocompatible ZnONPs with significant antibacterial potential. In conclusion, the strong antibacterial potential of *G. glabra*-mediated ZnONPs truly demands further investigation to completely understand their antimicrobial mechanism before considering them as a suitable alternative to conventional antibiotics.

## Author contributions

Krishna Kanta Samanta: writing – original draft, writing – review & editing, visualization, investigation. Manoj Kumar: writing – review & editing, resources. Himanshu Prasad Mamgain: investigation, formal analysis. Pritam Hait: data curation. Suvendu Manna: writing – review & editing, supervision. Bibhas Bhunia: writing – review & editing, writing – original draft. Soumen Basu: supervision, Jitendra Kumar Pandey: writing – review & editing, supervision. All authors approved the final version of this manuscript.

## Conflicts of interest

We have no conflicts to disclose.

## Supplementary Material

RA-015-D5RA05670E-s001

## Data Availability

The data supporting the findings of this study are available from the corresponding author upon resonable request. Supplementary information is available: providing details on the schematic elucidation for ZnONPs preparation, UV-Vis, TGA trace of *G. glabra* extract, and Zeta potential result. Additional information in tabular form, like a list of identified phytoconstituents, optimiztion using UV-Vis spectra, complete details of crystallite size calculation using Debye–Scherrer's, W-H, and Modified Scherrer's methods, and a comparison table of TGA analysis. See DOI: https://doi.org/10.1039/d5ra05670e.
