# Supplementary material for: Nature-inspired ZnO nanoparticles: unlocking the biomedical potential of Glycyrrhiza glabra-mediated green synthesis through in vitro and in silico approaches
Source: RSC Adv. 2025 Sep 26;15(42):35598–616. doi: 10.1039/d5ra05670e (PMC12466927; doi:10.1039/d5ra05670e)
Supplement: RA-015-D5RA05670E-s001 [file RA-015-D5RA05670E-s001.pdf]

Supplementary Information (SI) for RSC Advances.
This journal is © The Royal Society of Chemistry 2025

Nature-Inspired ZnO Nanoparticles: Unlocking the Biomedical Potential of *Glycyrrhiza glabra*-Mediated Green Synthesis through In-vitro and In-silico Approaches

Krishna Kanta Samanta [a], Manoj Kumar [b], Himanshu Prasad Mamgain [c], Pritam Hait [d], Suvendu Manna [e], Bibhas Bhunia [f], Soumen Basu [d] *, Jitendra K. Pandey [g] *,

[a] Department of Chemistry, School of Advanced Engineering, UPES, Dehradun, Uttarakhand, India (krishnakanta0096@gmail.com)

[b] Department of Biotechnology, School of Health Sciences and Technology, UPES, Dehradun, Uttarakhand, India (manoj.110762@stu.upes.ac.in)

[c] Department of Physics, School of Advanced Engineering, UPES, Dehradun, Uttarakhand, India (himanshuhm1111@gmail.com)

[d] Department of Chemistry, School of Chemistry & Biochemistry, Thapar Institute of Engineering and Technology, Patiala, Punjab, India (pritam.kr96@gmail.com) (soumen.basu@thapar.edu)

[e] Department of Microbiology, Sustainability Cluster, UPES, Dehradun, Uttarakhand, India (smanna@ddn.upes.ac.in)

[f] Department of Life Sciences, Parul Institute of Applied Sciences, Parul University, Vadodara, Gujrat 391760, India (bibhasmicro@gmail.com)

[g] HILL Institute, UPES, Dehradun, Uttarakhand, India (jeetusnu@gmail.com)

*Corresponding author: Prof. Soumen Basu (soumen.basu@thapar.edu)
Prof. Jitendra K. Pandey (jeetusnu@gmail.com)

Fig. S1: a) Schematic elucidation for the preparation of ZnONPs using the green method, where *G. glabra* (mulethi) extract was used as reducing, capping/stabilizing agents, and **b)** Collection of samples at different reaction times, i.e., 30, 60, 90, and 120 minutes for UV-visible analysis.

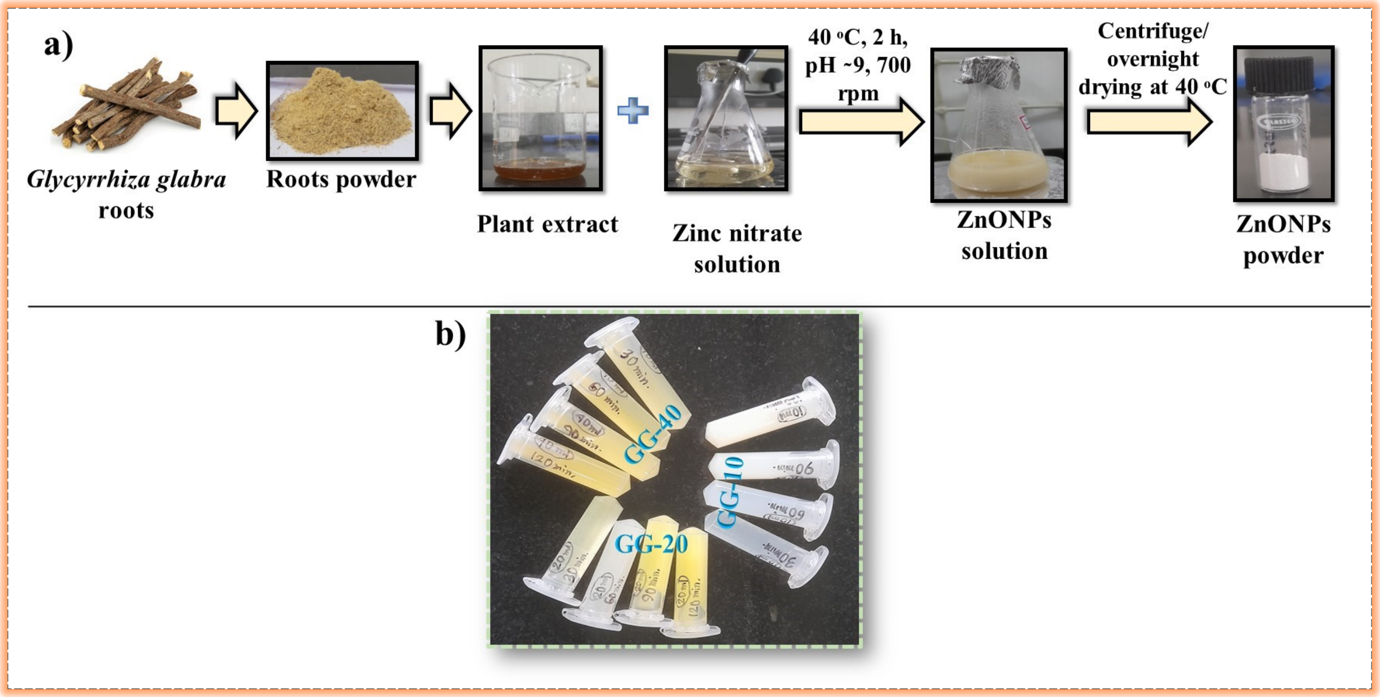

Fig. S2: UV-visible spectra of chemically synthesized ZnONPs (Chem.-ZnO). Band gap of Chem.-ZnO in the inserted figure.

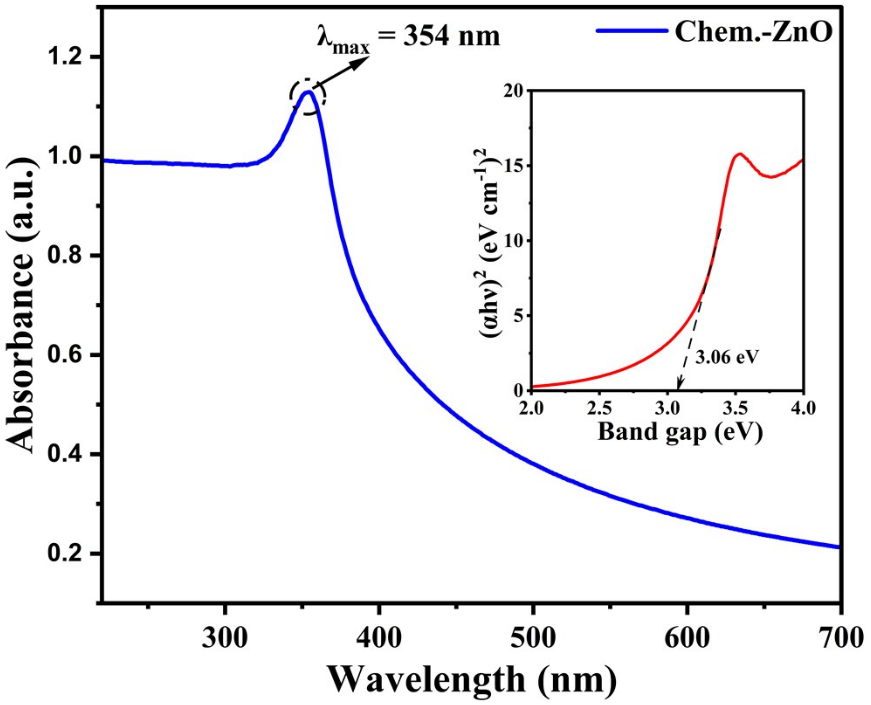

Fig. S3: TGA trace of *G. glabra* extract alone.

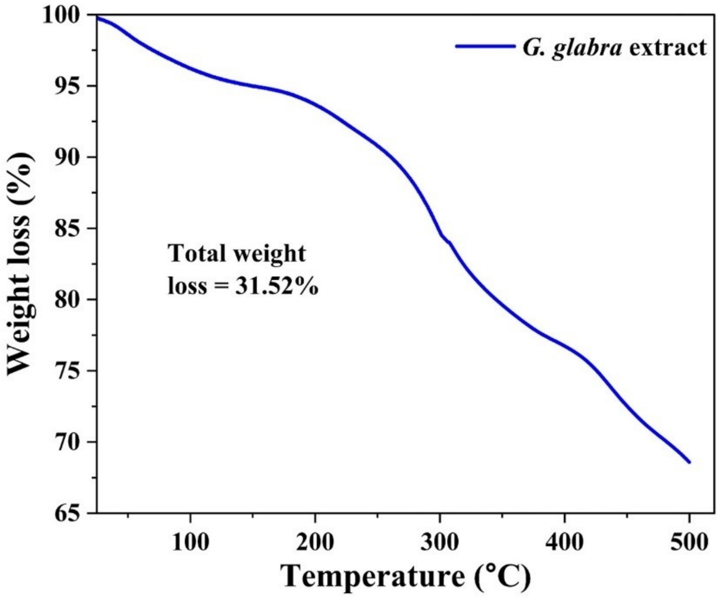

Fig. S4: Zeta potential analysis of (a) GG-10, (b) GG-20, (c) GG-40, and (d) Chem.-ZnO.

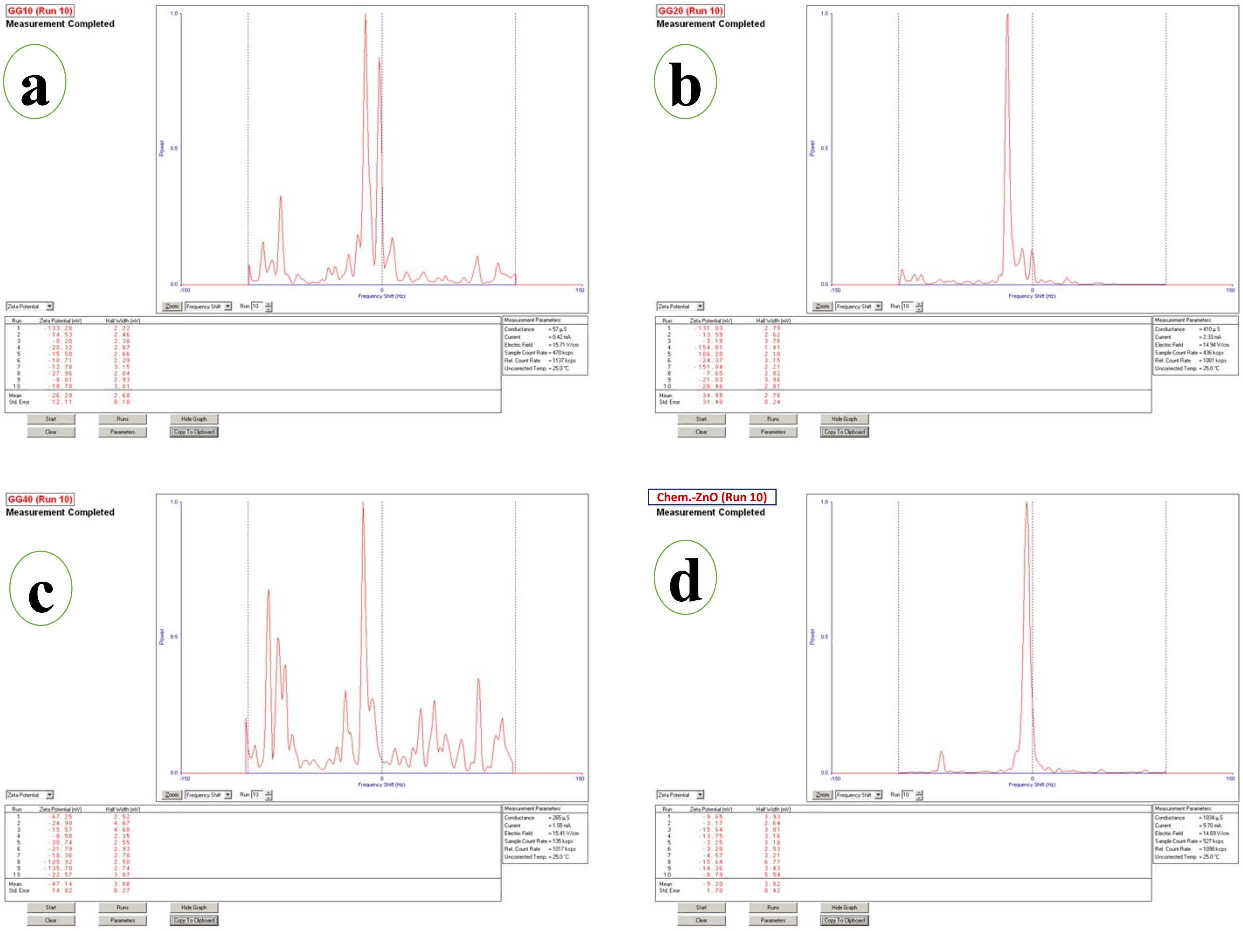

Table S1: List of identified phytoconstituents from methanolic extract of *G. glabra* root.

S/No.	Retention time (min)	% Area	Compound name	Structure
**1.**	5.57	0.90	Pentyl glycolate	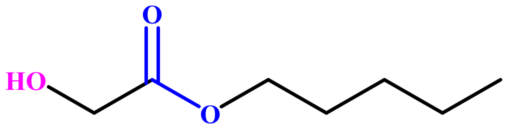
**2.**	7.03	0.90	Imidazole-5-carboxylic amide, N-methyl-	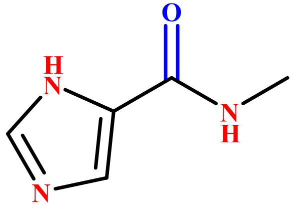
**3.**	21.67	11.41	n-Hexadecanoic acid	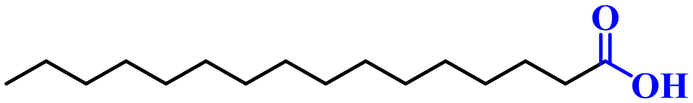
**4.**	25.10	35.23	9,12-Octadecadienoic acid (Z,Z)-	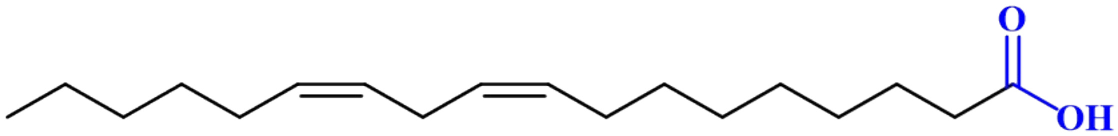

Table S2: Details for optimizing *G. glabra* root mediated ZnONPs synthesis.

S/ No.	Volume of Plant extract (10%w/v)	Volume of salt Solution (~ 0.1M)	Reaction time (min)	Color of the nanoparticle solution	Samples name	Absorbance at λmax (a.u.)
1	10 ml	90 ml	30	White		0.20
			60	White		0.41
			**90**	Milky white	GG-10	**0.58**
			120	Milky white		0.52
2	20 ml	90 ml	30	White		0.20
			60	Milky white		0.56
			90	Yellow	GG-20	0.74
			**120**	Yellow		**0.93**
3	40 ml	90 ml	30	Light yellow		0.75
			60	Yellow		0.96
			90	Yellow	GG-40	1.09
			**120**	Yellow		**1.16**

Optimized reaction time with corresponding absorbance is present in bold.

Table S3: Calculation of average crystallite size using **Debye-scherrer's method**.

2θ (degree)			FWHM (degree)			Diffraction peaks	Crystallite sizes (D) (nm)		
**GG-10**	**GG-20**	**GG-40**	**GG-10**	**GG-20**	**GG-40**		**GG-10**	**GG-20**	**GG-40**
31.75559	31.75	31.77	0.47587	0.59889	0.72876	100	18.13673	14.411	11.84345
34.39491	34.4326	34.44	0.31092	0.47823	1.49532	002	27.94896	18.17279	5.812099
36.2462	36.23679	36.22	0.49412	0.54758	1.51523	101	17.67719	15.95095	5.764142
47.51705	46.55	47.08465	0.50302	0.70916	0.89	102	18.03033	12.74241	10.17378
56.59356	56.53345	56.42451	0.4791	0.89932	0.74141	110	19.67672	10.47954	12.70505
62.83217	62.7319	62.6395	0.53526	0.84393	1.60344	103	18.17082	11.51864	6.059572
67.97277	67.89	68.06665	0.78252	1.0233	2.45396	112	12.79205	9.777355	4.081387
				Average crystallite size (nm)			**18.92**	**12.74**	**10.17**

Table S4: Calculations for the **W-H plot.**

Theta (radians)			FWHM (radians)			GG-10		GG-20		GG-40	
**GG-10**	**GG-20**	**GG-40**	**GG-10**	**GG-20**	**GG-40**	**$\beta_{hkl}\cos\theta$**	**$4\sin\theta$**	**$\beta_{hkl}\cos\theta$**	**$4\sin\theta$**	**$\beta_{hkl}\cos\theta$**	**$4\sin\theta$**
0.276271967	0.2769235	0.27709794	0.008301076	0.010447037	0.012712489	0.007985	1.093778	0.010049	1.093591	0.012228	1.094262
0.299330929	0.300321137	0.30038568	0.005423688	0.008342244	0.026084362	0.005181	1.182052	0.007969	1.183308	0.024916	1.183555
0.315385078	0.316057282	0.31591084	0.008619429	0.009551986	0.026431672	0.008192	1.243598	0.009079	1.243286	0.025124	1.242729
0.413742461	0.4060091	0.410672317	0.008774681	0.012370587	0.01552516	0.008032	1.610723	0.011365	1.579784	0.014234	1.596903
0.492798931	0.493084751	0.492134576	0.00835742	0.015687738	0.012933156	0.00736	1.895229	0.013819	1.893382	0.011398	1.890033
0.547254476	0.547147632	0.546341719	0.009337075	0.014721515	0.027970407	0.00797	2.084	0.012572	2.081014	0.023899	2.07826
0.592042731	0.59213658	0.593677321	0.013650279	0.017850445	0.042806878	0.011321	2.234936	0.014811	2.23254	0.035482	2.237652

Table S5: Calculations for the **Modified Scherrer's** plot.

Theta (radians)			FWHM (radians)			GG-10		GG-20		GG-40	
**GG-10**	**GG-20**	**GG-40**	**GG-10**	**GG-20**	**GG-40**	**ln (1/cosθ)**	**ln β**	**ln (1/cosθ)**	**ln β**	**ln (1/cosθ)**	**ln β**
0.276271967	0.2769235	0.27709794	0.008301076	0.010447037	0.012712489	0.0388575	-4.79137	0.0388436	-4.56144	0.0388932	-4.36517
0.299330929	0.300321137	0.30038568	0.005423688	0.008342244	0.026084362	0.0456893	-5.21698	0.0457911	-4.78642	0.045811	-3.64642
0.315385078	0.316057282	0.31591084	0.008619429	0.009551986	0.026431672	0.0508273	-4.75374	0.0508005	-4.65101	0.0507526	-3.63319
0.413742461	0.4060091	0.410672317	0.008774681	0.012370587	0.01552516	0.0884592	-4.73588	0.0847909	-4.39243	0.0868085	-4.16529
0.492798931	0.493084751	0.492134576	0.00835742	0.015687738	0.012933156	0.1271193	-4.78461	0.1268374	-4.15488	0.1263274	-4.34796
0.547254476	0.547147632	0.546341719	0.009337075	0.014721515	0.027970407	0.1583434	-4.67376	0.1578101	-4.21845	0.1573196	-3.57661
0.592042731	0.59213658	0.593677321	0.013650279	0.017850445	0.042806878	0.1871167	-4.294	0.1866307	-4.02573	0.1876689	-3.15106

Table S6: Three-stage weight loss by three samples from 25-800 ˚C.

Stage	Temperature (˚C)	Δm (%)		
		GG-10	GG-20	GG-40
1st	25-225	2.39	3.51	5.33
2nd	225-502	3.93	5.44	7.57
3rd	502-800	5.74	9.29	11.74

Table S7: Comparison of thermogravimetric analysis (TGA) with earlier published work based on total weight loss.

S/No.	Coating agent used	NPs	Temperature range (°C)	Heating rate (°C/min)	Total weight loss (%)	References
1	No coating agent	ZnO (bare)	R.T. to 400	Not mention	3.6	[1]
2	*Limonium pruinosum (L.)*	ZnO	R.T. to 1000	10	29.08	[2]
3	*Lippia adoensis*	ZnO	R.T. to 1000	10	36.27	[3]
4	*Peltophorum pterocarpum* leaf	ZnO	R.T. to 800	10	12	[4]
6	*Rubus fairholmianus* Root	ZnO	R.T. to 1000	10	7	[6]
7	Cow dung	ZnO	R.T. to 700	Not mention	21	[7]
8	*G. glabra* root extract	ZnO (GG-10)	R.T. to 800	10	12.06	**This work**
		ZnO (GG-20)	R.T. to 800	10	18.24	**This work**
		ZnO (GG-40)	R.T. to 800	10	24.64	**This work**

R.T. means room temperature

References

[1] H. Ghaffari *et al.*, “Inhibition of H1N1 influenza virus infection by zinc oxide nanoparticles: Another emerging application of nanomedicine,” *J. Biomed. Sci.*, vol. 26, no. 1, pp. 1–10, 2019, doi: 10.1186/s12929-019-0563-4.

[2] B. Naiel, M. Fawzy, M. W. A. Halmy, and A. E. D. Mahmoud, “Green synthesis of zinc oxide nanoparticles using Sea Lavender (Limonium pruinosum L. Chaz.) extract: characterization, evaluation of anti-skin cancer, antimicrobial and antioxidant potentials,” *Sci. Rep.*, vol. 12, no. 1, pp. 1–12, 2022, doi: 10.1038/s41598-022-24805-2.

[3] M. G. Demissie, F. K. Sabir, G. D. Edossa, and B. A. Gonfa, “Synthesis of Zinc Oxide Nanoparticles Using Leaf Extract of Lippia adoensis (Koseret) and Evaluation of Its Antibacterial Activity,” *J. Chem.*, vol. 2020, 2020, doi: 10.1155/2020/7459042.

[4] S. Pai, H. Sridevi, T. Varadavenkatesan, and R. Vinayagam, “Optik Photocatalytic zinc oxide nanoparticles synthesis using Peltophorum pterocarpum leaf extract and their characterization,” *Opt. - Int. J. Light Electron Opt.*, vol. 185, no. February, pp. 248–255, 2019, doi: 10.1016/j.ijleo.2019.03.101.

[5] “Green Synthesis of Zinc Oxide Nanoparticles Using Garlic Skin Extract and Its Characterization,” vol. 10, no. 1, pp. 20–27, 2020, doi: 10.22052/JNS.2020.01.003.

[6] N. K. Rajendran, B. P. George, N. N. Houreld, and H. Abrahamse, “Synthesis of zinc oxide nanoparticles using rubus fairholmianus root extract and their activity against pathogenic bacteria,” *Molecules*, vol. 26, no. 10, 2021, doi: 10.3390/molecules26103029.

[7] Z. Javed, G. D. Tripathi, M. Mishra, M. Gattupalli, and K. Dashora, “Cow dung extract mediated green synthesis of zinc oxide nanoparticles for agricultural applications,” *Sci. Rep.*, vol. 12, no. 1, pp. 1–12, 2022, doi: 10.1038/s41598-022-22099-y.
